# Network Pharmacology and Molecular Docking Study of Yupingfeng Powder in the Treatment of Allergic Diseases

**DOI:** 10.1155/2022/1323744

**Published:** 2022-07-09

**Authors:** Minye Qu, Wenhua Tao, Jian Ma

**Affiliations:** ^1^Department of Traditional Chinese Medicine, First Clinical Medical College, Jiangsu University, Zhenjiang, China; ^2^Department of Warm Disease, Basic Medical College, Nanjing University of Chinese Medicine, Nanjing, China

## Abstract

**Objective:**

To explore the potential mechanisms of Yupingfeng Powder (YPFP) in the treatment of allergic diseases by using network pharmacology and molecular docking technology.

**Methods:**

The active components and targets of YPFP were screened by the TCMSP database. The targets associated with atopic dermatitis, asthma, allergic rhinitis, and food allergy were obtained from GeneCards and OMIM databases, respectively. The intersection of the above disease-related targets was identified as allergy-related targets. Then, allergy-related targets and YPFP-related targets were crossed to obtain the potential targets of YPFP for allergy treatment. A protein-protein-interaction (PPI) network and a drug-target-disease topology network were constructed to screen hub targets and key ingredients. Next, GO and KEGG pathway enrichment analyses were performed separately on the potential targets and hub targets to identify the biological processes and signaling pathways involved. Finally, molecular docking was conducted to verify the binding affinity between key ingredients and hub targets.

**Results:**

In this study, 45 active ingredients were identified from YPFP, and 48 allergy-related targets were predicted by network pharmacology. IL6, TNF, IL1B, PTGS2, CXCL8, JUN, CCL2, IL10, IFNG, and IL4 were screened as hub targets by the PPI network. However, quercetin, kaempferol, wogonin, formononetin, and 7-O-methylisomucronulatol were identified as key ingredients by the drug-target-disease topological network. GO and KEGG pathway enrichment analysis indicated that the therapeutic effect of YPFP on allergy involved multiple biological processes and signaling pathways, including positive regulation of fever generation, positive regulation of neuroinflammatory response, vascular endothelial growth factor production, negative regulation of cytokine production involved in immune response, positive regulation of mononuclear cell migration, type 2 immune response, and negative regulation of lipid storage. Molecular docking verified that all the key ingredients had good binding affinity with hub targets.

**Conclusion:**

This study revealed the key ingredients, hub targets, and potential mechanisms of YPFP antiallergy, and these data can provide some theoretical basis for subsequent allergy treatment and drug development.

## 1. Introduction

Allergies are a class of recurrent, chronic inflammatory diseases. Over the past few decades, the incidence of allergic diseases has continued to rise, especially in developing countries [1]. Many cross-sectional and longitudinal studies have shown that the occurrence of allergic diseases follows a natural process, including the time evolution from preschool atopic dermatitis and food allergy to school-age asthma and allergic rhinitis, as well as the spatial evolution from the skin and gastrointestinal tract to respiratory tract [2, 3]. With the consensus of the natural process of allergy, the whole-course management of allergy has been advocated in recent years [4]. However, currently available therapeutic approaches against allergy, such as glucocorticoids, antihistamines, leukotriene inhibitors, and oral immunotherapy can only temporarily control symptoms, but cannot effectively control disease recurrence or inhibit the atopic progression [5, 6]. In addition, long-term use of these drugs has certain side effects [6–8].

Traditional Chinese medicine (TCM) has a long history of treating allergic diseases. The “holistic concept” of TCM emphasizes the correlation between the various components of the human body, which coincides with allergy whole-course management concept of modern medicine. Due to the good clinical efficacy and few side effects, TCM treatment of allergy is widely used in China and has been gradually accepted worldwide as a major treatment for complementary and alternative medicine [9, 10].

Yupingfeng Powder (YPFP) originated from the ancient Chinese medicine book “Danxi Xinfa,” which is composed of three Chinese herbs: Huangqi, Fangfeng, and Baizhu. In allergic diseases, YPFP shows good effects on both improving symptoms and controlling recurrence [11–13]. It has been confirmed that the glycoside extract of YPFP exerts antiinflammatory and immunological functions by regulating T lymphocyte differentiation subtypes [14]. The Huangqi-Fangfeng drug pair prevents allergic airway remodeling by inhibiting the epithelial-mesenchymal transformation process via regulating the epithelial-derived TGF-*β*1 [15]. The polysaccharide isolated from Baizhu enhances immunity by promoting the proliferation of CD4, CD8, and Treg cells [16, 17]. However, the TCM compound has the characteristics of multiple components, multiple targets, and multiple pathways. The pharmacodynamic components, effective targets, and molecular mechanisms of YPFP on allergy remain to be further studied.

**Figure 1:**
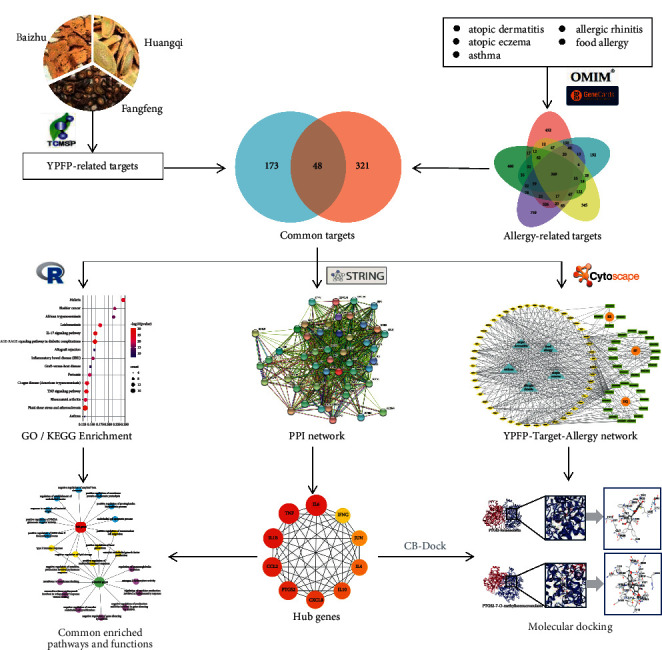
Workflow of this study.

With the rapid development of system biology and system pharmacology, network pharmacology has transformed the drug research models from “single disease-single target” to “multiple disease-multiple target”, which provides a new way for us to systematically study the pharmacodynamic mechanism of TCM compounds [18, 19]. Therefore, in this study, network pharmacology was conducted to predict the active ingredients of YPFP as well as its targets and pathways for allergy treatment. In addition, molecular docking techniques were used to verify the interactions between ingredients and targets. This study aimed to provide a theoretical basis for the potential mechanism of YPFP antiallergy. The workflow is shown in Figure 1.

## 2. Methods

### 2.1. Active Ingredient Screening of YPFP

The active ingredients of YPFP were obtained from the Traditional Chinese Medicine Systems Pharmacology Database and Analysis Platform (TCMSP) (https://tcmspw.com/tcmsp.php), which is a unique TCM systematic pharmacology platform that integrates TCM active ingredients, potential targets, associated diseases, and pharmacokinetic data. Using “Huangqi,” “Fangfeng,” and “Baizhu” as keywords, oral bioavailability (OB) ≥30% and drug-likeness (DL) ≥0.18 as screening conditions, a total of 45 active ingredients were obtained. Download the molecular structures of the screened active ingredients to prepare for subsequent molecular docking.

### 2.2. Target Prediction of Active Ingredients

Targets corresponding to the active ingredients of YPFP were predicted from TCMSP and translated into gene names using UniProtKB (https://www.uniprot.org). After removing duplicate genes, 221 YPFP-related target genes were obtained.

### 2.3. Allergy-Related Target Gene Collection

Allergy-related target genes were searched for the keywords “atopic dermatitis,” “atopic eczema,” “asthma,” “allergic rhinitis,” and “food allergy” by GeneCards (https://www.genecards.org) and OMIM (https://www.omim.org). The data from the two databases were merged to remove the duplicates, and the related genes for each keyword were obtained. Cross the five keyword-related genes by “UpSet” and “VennDiagram” *R* package to obtain allergy-related target genes.

### 2.4. Clustering of YPFP and Allergy-Related Target Genes

Venny2.1 (https://bioinfogp.cnb.csic.es/tools/venny) was used to cluster YPFP-related target genes and allergy-related target genes, and the obtained overlaps were considered as potential target genes of YPFP therapy for allergy.

### 2.5. PPI Network Construction and Hub Gene Extraction

The PPI network was plotted by entering the potential target genes into the STRING database (https://www.string-db.org), with the species set as “*Homo sapiens*” and a confidence score ≥0.4. Each node in the network represents a protein, and the connection lines between nodes represent functional associations. The relevant results were exported in TSV format and extracted by Cytoscape v_3.9.1, and the top 10 hub genes were identified by the McCreight (MCC) method using the “Cytohubba” plug-in.

### 2.6. Construction of YPFP-Target-Allergy Topological Network

Cytoscape v_3.9.1 was used to analyze and visualize complex networks among herbs, active ingredients, potential targets, and diseases. By analyzing the network topology parameters, the key ingredients were screened.

### 2.7. GO and KEGG Pathway Enrichment Analysis

KOBAS 3.0 (https://KOBAS.cbi.pku.edu.cn) was used for the KEGG and GO analyses of the potential genes and hub genes, respectively. The enrichment degree was calculated according to the input number/background number, with *P* value ≤ 0.05 and an input number ≥2 as the inclusion criteria. Based on descending order of enrichment degree, the top 15 KEGG pathway and top 15 GO items of potential genes and hub genes were separately plotted as bubble diagrams by the “ggplot2” *R* package. Then Cytoscape v_3.9.1 was used to construct topological networks to identify the common biological functions and signaling pathways of hub genes and potential genes in the top 15 GO and KEGG results. In addition, the “ggalluvial” *R* package was used to draw the Sankey diagram of hub genes and enriched signal pathways. KEGG Mapper (https://www.genome.jp/kegg/mapper) is a collection of tools for KEGG mapping, which visualizes the relevant pathways of acquired genes through “Search Pathway” and “Search & Color Pathway” tools. We input potential genes into KEGG Mapper and set different colors (the hub genes are red, the remaining potential genes are green), and then got the most relevant signaling pathway and displayed them.

### 2.8. Molecular Docking of Hub Targets and Key Ingredients

To verify the binding affinity of the hub targets of the PPI network to the key ingredients of the topological network, we performed molecular docking. CB-Dock is an online molecular docking website (https://clab.labshare.cn/cb-dock/php/). It predicts the binding region of a given protein, calculates the center and size using a curvation-based cavity detection method, connects with ligand binding sites queried by Autodock Vina, and then sorts binding patterns based on Vina scores and provides interactive 3D visualization of binding patterns. The 3D structures of the hub targets obtained by RSCB PDB and the 2D structures of the key ingredients obtained by TCMSP were uploaded to the CB-Dock website. After automatically identifying cavity size, calculating center and size, molecular docking, and configurational scoring were performed. Cavity size is the protein-ligand interaction interface predicted by CB-Dock based on the concave surface of protein [20]. Generally, the larger the cavity size is, the higher the affinity between protein and ligand will be [20]. The center and size of the docking box are evaluated according to the cavity center, cavity size, and ligand size, which are key parameters in the process of molecular docking [21]. The binding affinity was evaluated according to the Vina score of the docking results. The greater the absolute value of the Vina score, the more stable the docking module is. The 3D conformation of the target-ingredient docking with a Vina score ≤ −8.5 kcal/mol was downloaded for display.

## 3. Results

### 3.1. Active Ingredients and Targets Screening of YPFP

According to OB ≥ 30% and DL ≥ 0.18, a total of 45 active ingredients of YPFP were screened from the TCMSP database, including 20 from Huangqi, 18 from Fangfeng, and 7 from Baizhu (Table 1). By TCMSP target prediction and UniProt gene name transformation, 221 YPFP-related target genes were obtained after removing duplicates (Supplementary Table S1).

### 3.2. Allergy-Related Target Gene Collection

**Figure 2:**
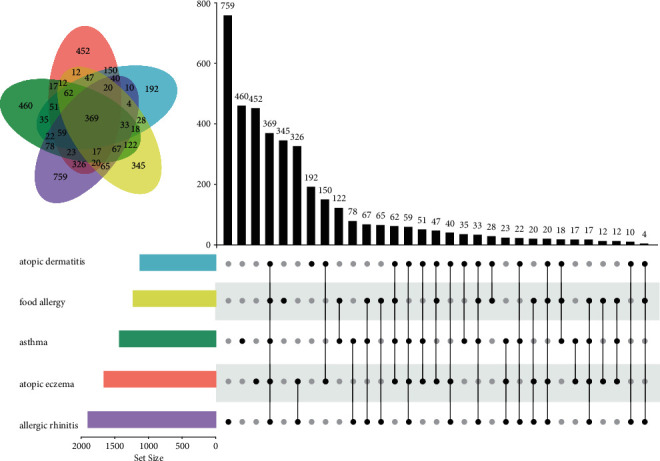
Allergy-related target genes obtained by clustering target genes corresponding to “atopic dermatitis,” “atopic eczema,” “asthma,” “allergic rhinitis,” and “food allergy.”

The GeneCards and OMIM databases were searched with the keywords “atopic dermatitis,” “atopic eczema,” “asthma,” “allergic rhinitis,” and “food allergy.” When the search results of the two databases were combined (if relevance score >1, duplicates were removed), the target genes corresponding to the 5 keywords were 1140, 1677, 1445, 1912, and 1241, respectively (Supplementary Table S2). By clustering the genes associated with the 5 keywords, 369 shared genes were obtained as allergy-related target genes (Figure 2).

### 3.3. Potential Target Genes and PPI Network of YPFP Therapy for Allergy

**Figure 3:**
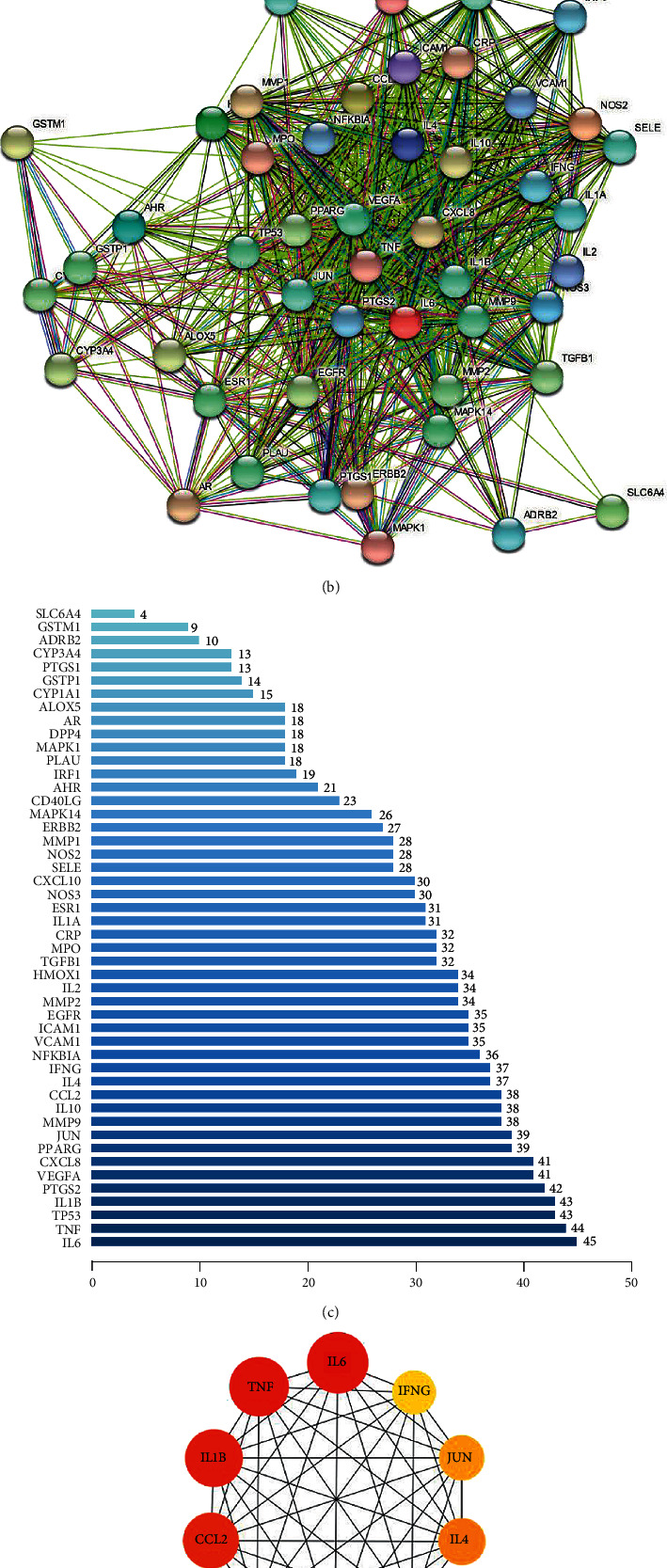
Potential target genes and PPI network of YPFP therapy for allergy. (a) The Venny results of potential target genes of YPFP therapy for allergy. (b) The PPI network of 48 potential genes. (c) Count and list of the genes of the PPI network. (d) Hub genes from the PPI network.

YPFP-related target genes and allergy-related target genes were intersected by Venny2.1 to obtain 48 potential genes (Figure 3(a)). These 48 potential genes were imported into the STRING database to obtain the PPI network (Figure 3(b)). According to the node degree of each protein in the network, the importance priority was analyzed and a bar graph was drawn for visualization (Figure 3(c), Supplementary Table S3). In order to screen hub genes from the potential genes, PPI network files were imported into Cytoscape v_3.9.1, and the top 10 genes were identified as hub genes by the MCC method of the “CytoHubba” plug-in, including IL6, TNF, IL1B, PTGS2, CXCL8, JUN, CCL2, IL10, IFNG, and IL4 (Figure 3(d), Supplementary Table S4).

### 3.4. YPFP-Target-Allergy Topological Network

**Figure 4:**
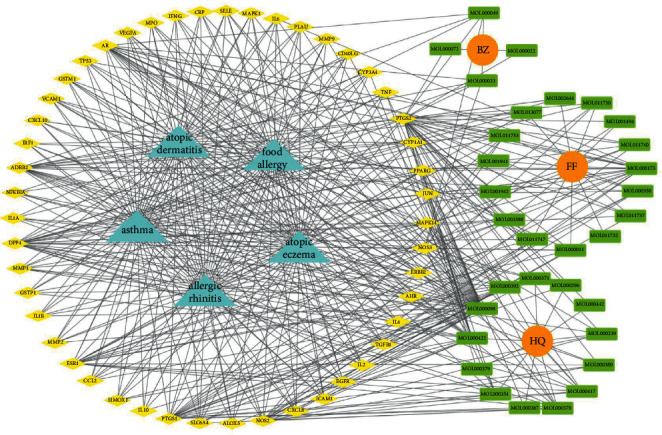
YPFP-Target-Allergy network. The orange circles represent drugs; the green squares represent active ingredients; the yellow diamonds represent potential targets; and the blue triangles represent the diseases. The line between two nodes represents the interaction.

Cytoscape v_3.9.1 was used to construct a topological network to visualize the complex relationship between YPFP active ingredients and allergy-related targets. The YPFP-Target-Allergy network consists of 88 nodes and 478 interactions. These nodes were 32 YPFP active ingredients, 48 potential targets, 5 keywords of allergy-related diseases, and 3 herbs (Figure 4). The active ingredients were sorted according to the descending order of degree value, and the top 5 were predicted to be the key ingredients, including quercetin, kaempferol, wogonin, formononetin, and 7-O-methylisomucronulatol (Table 2).

### 3.5. GO and KEGG Pathway Enrichment Analysis of Potential Genes and Hub Genes

**Figure 5:**
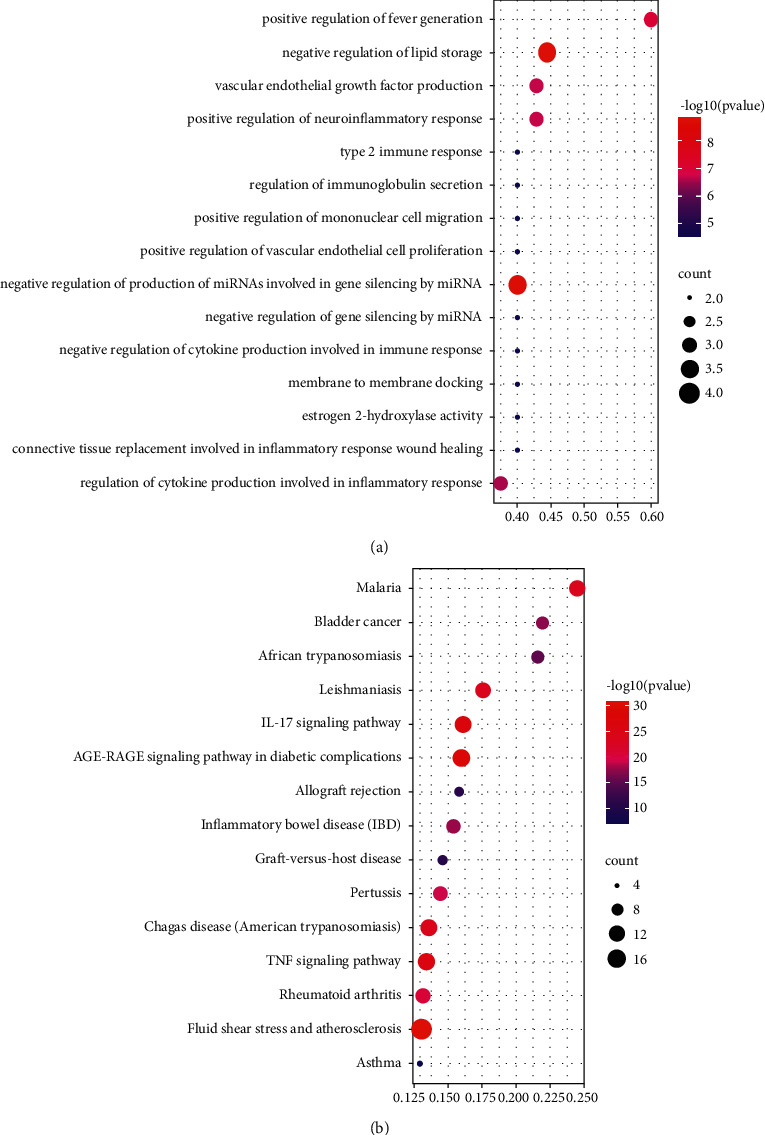
The top 15 remarkably enriched GO and KEGG pathway analyses of potential genes. (a) The top 15 remarkably enriched GO analyses for biological function of potential target genes of YPFP in allergy. (b) The top 15 remarkably enriched KEGG analyses for signaling pathway of potential target genes of YPFP in allergy.

To explore the regulatory mechanisms of potential target genes on allergies, we conducted GO and KEGG enrichment analyses and selected the top 15 remarkably enriched biological functions and signal pathways to generate bubble diagrams, respectively (Figure 5).

**Figure 6:**
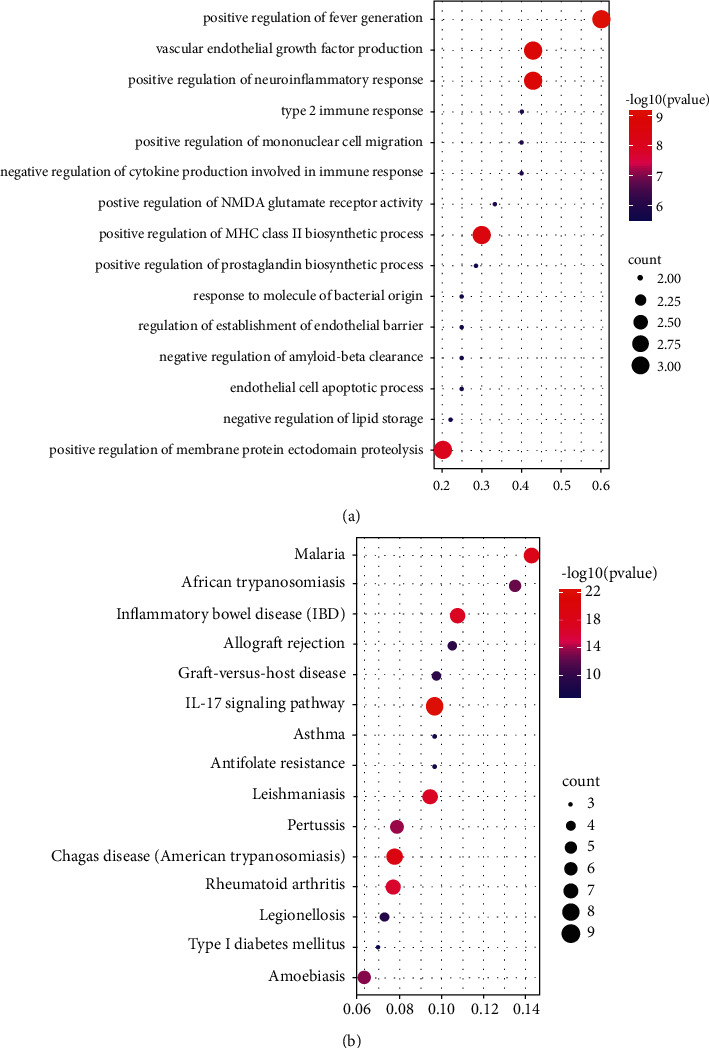
The top 15 remarkably enriched GO and KEGG pathway analyses of hub genes. (a) The top 15 remarkably enriched GO analyses for biological function of hub genes of YPFP in allergy. (b) The top 15 remarkably enriched KEGG analyses for signaling pathway of hub genes of YPFP in allergy.

**Figure 7:**
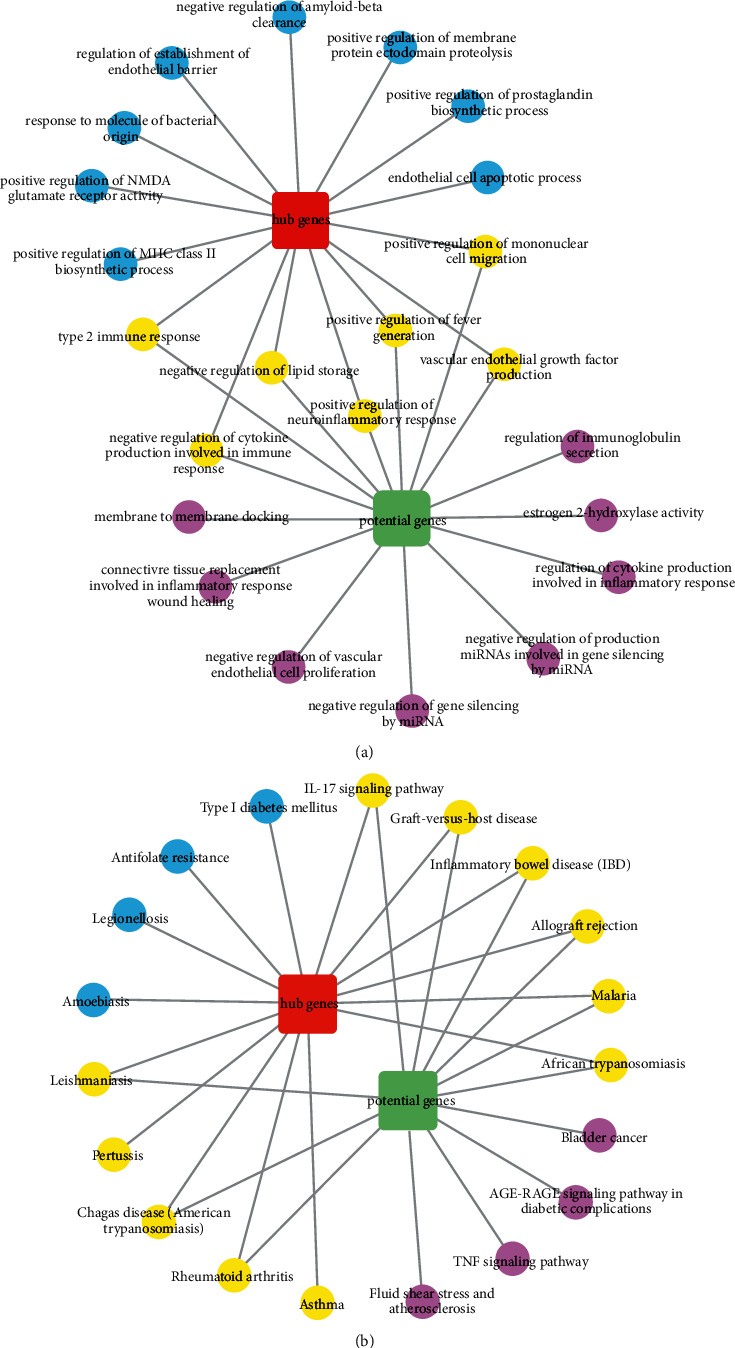
Co-enrichment functions and pathways of hub genes and potential genes in the top 15 GO and KEGG analyses. (a) The common enriched functions of hub genes and potential genes in the top 15 GO analyses. (b) The common enriched pathways of hub genes and potential genes in the top 15 KEGG analyses. The red square represents hub genes; the green square represents potential genes; the blue circles represent biological functions or pathways enriched by hub genes; the purple circles represent biological functions or pathways enriched by potential genes; and the yellow circles represent co-enrichment of biological functions or pathways of hub genes and potential genes.

To further verify the dominant role of hub genes in potential genes, we also performed GO and KEGG enrichment analyses on hub genes and compared the results with those of potential genes. The GO and KEGG enrichment results of the top 15 hub genes were represented by bubble diagrams (Figure 6). In addition, the common enriched pathways and functions of hub genes and potential genes in the top 15 GO and KEGG analyses were displayed by topological networks (Figure 7).

GO enrichment analysis of hub genes and potential genes showed that 7 of the top 15 biological functions with the highest enrichment were identical, such as positive regulation of fever generation, positive regulation of neuroinflammatory response, vascular endothelial growth factor production, negative regulation of cytokine production involved in immune response, positive regulation of mononuclear cell migration, type 2 immune response, and negative regulation of lipid storage.

**Figure 8:**
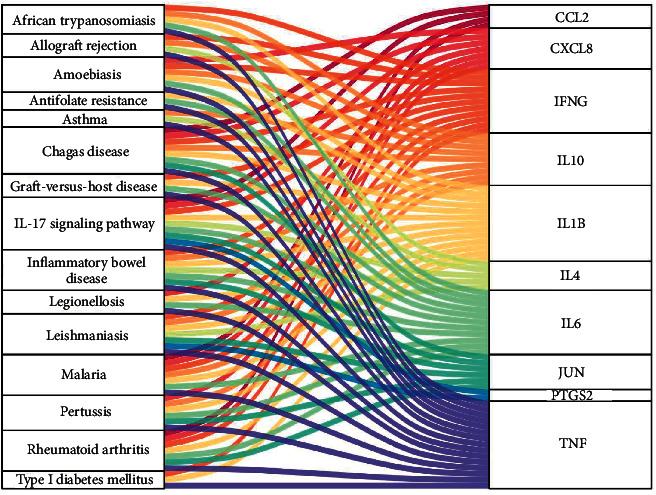
Sankey diagram of hub genes and enriched signaling pathways.

**Figure 9:**
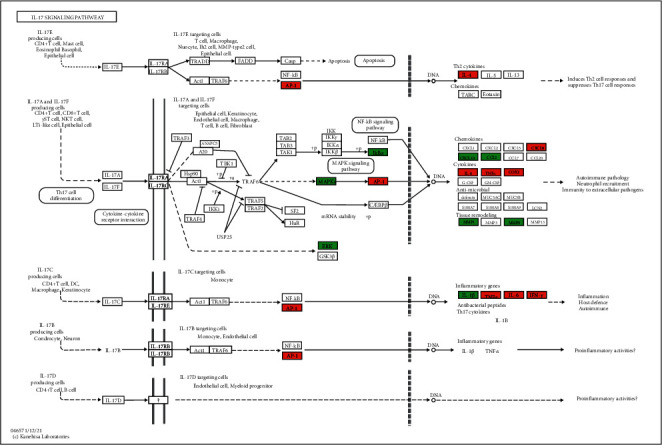
The IL-17 signaling pathway of YPFP-related genes in allergy. Arrows indicate upstream and downstream relationships between genes. The colored ones are YPFP target genes in the network, where red are the hub genes and green are the potential genes.

KEGG enrichment analysis showed that 11 of the top 15 signaling pathways with significant enrichment of hub genes and potential genes were the same, including malaria, African trypanosomiasis, inflammatory bowel disease (IBD), allograft rejection, graft-versus-host disease, IL-17 signaling pathway, asthma, leishmaniasis, pertussis, chagas disease (American trypanosomiasis), and rheumatoid arthritis. Additionally, the Sankey diagram showed that most hub genes were enriched in the IL-17 pathway. The Sankey diagram and signal pathway diagram are shown in Figures 8 and 9.

### 3.6. Molecular Docking Verification

**Figure 10:**
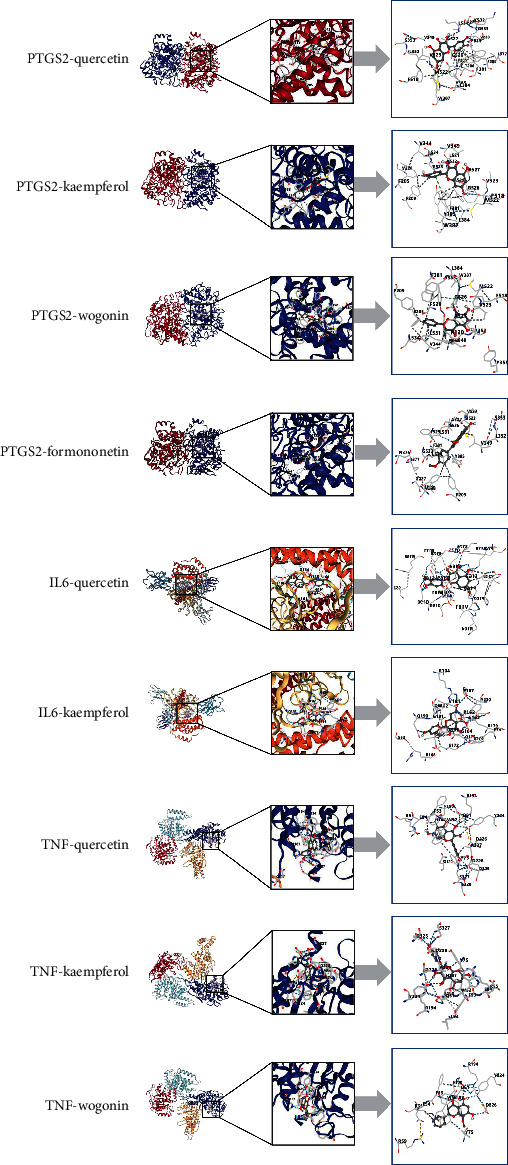
Molecular docking models with vina score less than −8.5 kcal/mol.

Molecular docking was used to evaluate the binding affinity between the hub targets of the PPI network and the key ingredients of the YPFP-Target-Allergy topological network. The results showed that the minimum vina score of each docking module was less than −5.0 kcal/mol, indicating a good binding affinity of the hub targets to the key ingredients (Table 3, Supplementary Table S5). To better demonstrate the docking patterns of the targets and ingredients, only the docking modules with a vina score of less than −8.5 kcal/mol were visualized (Figure 10).

## 4. Discussion

Atopic dermatitis (also known as atopic eczema), food allergies, asthma, and allergic rhinitis are the most common allergy-related diseases with similar epigenetic and physiopathological characteristics [22, 23]. Numerous experimental and clinical studies have shown that the poor prognosis of atopic dermatitis and food allergies in early life greatly exacerbates the occurrence of later asthma and allergic rhinitis [1–4, 24]. Although it is believed that the development of allergies is associated with genetic and environmental influences, the specific mechanisms remain unclear. Therefore, it is necessary to conduct joint research on multiple allergy-related diseases to explore their common core mechanisms.

TCM has a unique theoretical system for the treatment of allergies. According to the theory of TCM, “qi deficiency” and “wind evil” are the two pathogenic bases of allergic diseases. Therefore, invigorating qi and dispelling wind are the main treatment principles. YPFP, a TCM compound with the above dual effects, is considered an effective and safe complementary and alternative therapy for allergy. To reveal the underlying mechanism of YPFP for allergy treatment, we described the relationships between active ingredients, targets, and signaling pathways in combination with network pharmacology and molecular docking.

A PPI network was constructed with 48 genes co-expressed by allergy and YPFP. The 10 hub genes obtained by the PPI network all suggest the association of allergy and inflammatory response. IL6, TNF, CCL2, CXCL8, and IL1*β* were pro-inflammatory genes, while IL4, IL10, and IFNG were antiinflammatory genes. In the acute phase of inflammation, IL-6 is an important mediator of host immunity, but its sustained release can induce chronic inflammation or even a “cytokine storm” [25]. Mast cells (MC) are key participants in IgE lazy allergy [26]. In local reactions, MC-mediated CCL2 promotes basophil migration, and MC-mediated CXCL8 and IL-1*β* participate in the recruitment of neutrophils [27–29]. Besides, TNF, as an important derivative of MC, plays a key role in promoting the induction of adaptive immunity [26]. TH2 type adaptive immunity is a common immunological manifestation of allergy-related diseases [30]. TH2 polarization leads to dysregulation of T lymphocyte subsets, such as TH1/TH2 imbalance and TH2/Treg imbalance [31, 32]. As the main secreted cytokines of TH2, TH1, and Treg, the expressions of IL-4, IFN-*γ,* and IL-10 necessarily show corresponding changes [31, 32]. PTGS2 is a cyclooxygenase involved in pain, inflammation, and tumor formation. Recent studies have found that PTGS2 participates in the induction of neovascularization and the promotion of TH2 polarization [33–35]. Furthermore, Jun regulates inflammatory processes at the transcriptional level. It was confirmed that Jun/AP-1 activation plays a key role in the expression of pro-inflammatory molecules such as IL-1, IL-6, and TNF-A [36]. Thus, anaphylaxis is essentially a chronic inflammatory response involving multiple cells and mediators.

According to the screening conditions (OB ≥ 30%, DL ≥ 0.18), a total of 45 active ingredients and 221 targets related to YPFP were identified, indicating that YPFP exerts its pharmacological effects in the treatment of allergies via multiple targets. The top five active ingredients obtained by the YPFP-Target-Allergy topological network were quercetin, kaempferol, wogonin, formononetin, and 7-O-methylisomucronulatol. As for quercetin, existing studies have indicated that quercetin relieves asthmatic tracheal spasm by inhibiting the expression of MUC5AC in airway epithelial cells and improves allergic inflammation by regulating TH1/TH2 balance and reducing antigen-specific IgE antibodies [37, 38]. Kaempferol, a biological flavonoid, has been reported to block airway inflammation by interfering with TyK-STAT signaling in the airway epithelial cells of asthmatic mice [39]. Formononetin, a phytoestrogen extracted from Huangqi, has been demonstrated to ameliorate IL-13-induced inflammation and mucus formation in human nasal epithelial cells by activating the SIRT1/Nrf2 signaling pathway [40]. With regard to wogonin, it has been proved to reduce allergic airway inflammation by inducing eosinophil apoptosis and regulating T lymphocyte differentiation [41–43]. On the whole, YPFP is a compound with a multitarget therapeutic effect. The potential association of its active ingredients with allergies should be further investigated.

GO analysis revealed that the main biological processes of YPFP (significantly enriched by both potential genes and hub genes) included neuroinflammation, fever regulation, vascular endothelial growth, lipid storage, and inflammatory regulation. The pathogenesis of neuroinflammation in allergic diseases has long been reported. Patients with chronic allergic inflammation have similar patterns of neuroinflammatory gene expression, which may be associated with the outcome of neuroimmune interactions [44, 45]. Fever is regulated by hypothalamic temperature-sensitive neurons. Although infection is the primary cause of fever, endogenous heat sources such as IL-1*β*, TNF, IL-6, and PTGS2-induced prostaglandins also act on the thermoregulatory centers of allergic diseases [46, 47]. Obesity is thought to be a major environmental factor in promoting allergies [48, 49]. Adipose tissue synthesizes and secretes a variety of cytokines and adipokines such as IL-6, TNF, TGF-*β*, and leptin that drive hypoinflammatory responses and have been proven to contribute to the pathogenesis of allergy [50, 51]. Angiogenesis has been observed with the development of allergic inflammation. Vascular endothelial growth factor (VEGF) is one of the most important angiogenesis inducing factors, which contributes to allergic reactions by increasing vascular permeability and promoting local tissue fluid exudation [26, 52]. As for inflammatory regulation, specifically including negative regulation of cytokine production involved in immune response, positive regulation of mononuclear cell migration, and type 2 immune response, the relevant mechanisms have been mentioned in the previous paragraph. Therefore, it is speculated that the effect of YPFP on allergy may be related to the above biological processes.

KEGG pathway analysis revealed that YPFP treatment of allergy involves multiple signaling pathways. Examples include malaria, African trypanosomiasis, leishmaniasis, and Chagas disease (American trypanosomiasis), which are associated with parasitic infections. Numerous epidemiological and immunological studies have supported parasite expose-mediated host defense as a potential risk factor for allergy [53–55]. Inflammatory bowel disease (IBD) and rheumatoid arthritis are both autoimmune diseases, and pertussis is an acute respiratory infectious disease. It is currently believed that allergy is a nonlocalized chronic inflammatory disease, which can lead to systemic immune dysfunction and further increase the risk of irritable bowel syndrome, rheumatoid arthritis, and pertussis [56–59]. The IL-17 signaling pathway, which enriches most of the central targets, is presumed to be the most relevant pharmacodynamic pathway of YPFP. It has been confirmed that IL-17 plays a key role in host defense, allergic reactions, autoimmune diseases, and other inflammatory responses [60–64]. TH2 polarization-induced eosinophil infiltration was thought to be the core mechanism of hypersensitivity. However, IL-17 in combination with IL-23 amplifies the inflammatory effects of eosinophils by activating NF-*κ*B, ERK, and P38 MAPK signaling pathways [65]. Even in non-Th2 allergies, IL-17 induces airway epithelial cells to produce chemokines CXCL1 and CXCL8 to promote neutrophil recruitment while stimulating fibroblasts and macrophages to secrete cytokines such as GM-CSF, TNF, IL-1, IL-8, and IL-6 to enhance inflammatory responses [61, 66, 67]. Besides, corticosteroids are one of the effective means of allergy control, but steroid resistance is the main cause of refractory allergy [68]. Recent studies have suggested that IL-17 induces steroid hyporesponsiveness in obese asthmatic patients by mediating dysregulation of glucocorticoid receptor *α*/*β* [69]. In conclusion, IL-17 may be a key pathway for YPFP to exert its inflammatory regulatory role in allergic diseases. Investigation of the relevant drug-target action mechanism may be the direction of further exploration.

Molecular docking verified the good binding effect of key ingredients and hub targets. The binding scores of PTGS2 to quercetin, kaempferol, wogonin, and formononetin, IL-6 to kaempferol and formononetin, and TNF to kaempferol, wogonin, and formononetin were all less than −8.5 kcal/mol, indicating that the bound molecular structure was more stable. PTGS2 (COX-2) is an important mediator in promoting inflammation and tumor growth. Il-6 is involved in inducing the transition from acute to chronic inflammation. TNF-*α* mediates the inflammatory response in the early stages of anaphylaxis. Studies have shown that quercetin inhibits the production of COX-2 and delays angiogenesis by inactivating P300 histone acetyltransferase (HAT) [70]. Kaempferol targets STAT3 and NF-*κ*B signaling pathways to reduce inflammatory responses associated with PTGS2 expression [71]. Formononetin reduces the release of inflammatory mediators such as TNF-*α*, IL-1*β,* and IL-6 by inhibiting caspase-1 activity and regulating NF-*κ*B activation and translocation [72]. The above studies partially validated our findings.

However, there are still some limitations to this study. For example, the database information may be incomplete, and the interaction between active ingredients is not considered. Nonetheless, this study provides a new approach for further exploring the molecular mechanism of YPFP in allergies and a new idea for the study of targeted therapy mechanisms.

## 5. Conclusion

In this study, we combined network pharmacology and molecular docking technology to study the molecular mechanism of YPFP in the treatment of allergy. According to the research, YPFP plays an antiallergic role mainly through inhibiting inflammatory infiltration, regulating neuroinflammation, regulating fever, inhibiting vascular endothelial growth, and improving lipid storage, involving malaria, African trypanosomiasis, inflammatory bowel disease (IBD), allograft rejection, graft-versus-host disease, IL-17 signaling pathway, asthma, leishmaniasis, pertussis, Chagas disease (American Trypanosomiasis) and Rheumatoid arthritis and other signaling pathways. Quercetin, Kaempferol, Wogonin, Formononetin, and 7-o-methylisomucronulatol are the key active ingredients of YPFP. IL6, TNF, IL1*β*, PTGS2, CXCL8, JUN, CCL2, IL10, IFNG, and IL4 are the main therapeutic targets. This study indicates that YPFP has the characteristics of multicomponent, multitarget, and multipathway in allergy treatment, which provides more evidence for the further application of YPFP in allergy.

## Figures and Tables

**Table 1:** Characteristics of active ingredients in YPFP.

Herb name	Molecule ID	Molecule name	Molecule weight	OB (%)	DL
Huangqi (HQ)	MOL000211	Mairin	456.78	55.38	0.78
	MOL000239	Jaranol	314.31	50.83	0.29
	MOL000296	Hederagenin	414.79	36.91	0.75
	MOL000033	(3S, 8S, 9S, 10R, 13R, 14S, 17R)-10, 13-Dimethyl-17-[(2R, 5S)-5-propan-2-yloctan-2-yl]-2, 3, 4, 7, 8, 9, 11, 12, 14, 15, 16, 17-dodecahydro-1H-cyclopenta[a] phenanthren-3-ol	428.82	36.23	0.78
	MOL000354	Isorhamnetin	316.28	49.6	0.31
	MOL000371	3, 9-di-O-methylnissolin	314.36	53.74	0.48
	MOL000374	5′-Hydroxyiso-muronulatol-2′, 5′-di-O-glucoside	642.67	41.72	0.69
	MOL000378	7-O-Methylisomucronulatol	316.38	74.69	0.3
	MOL000379	9,10-Dimethoxypterocarpan-3-O-*β*-D-glucoside	462.49	36.74	0.92
	MOL000380	(6aR, 11aR)-9, 10-Dimethoxy-6a, 11a-dihydro-6H-benzofurano[3, 2-c] chromen-3-ol	300.33	64.26	0.42
	MOL000387	Bifendate	418.38	31.1	0.67
	MOL000392	Formononetin	268.28	69.67	0.21
	MOL000398	Isoflavanone	316.33	109.99	0.3
	MOL000417	Calycosin	284.28	47.75	0.24
	MOL000422	Kaempferol	286.25	41.88	0.24
	MOL000433	FA	441.45	68.96	0.71
	MOL000438	(3R)-3-(2-Hydroxy-3,4-dimethoxyphenyl)chroman-7-ol	302.35	67.67	0.26
	MOL000439	Isomucronulatol-7, 2′-di-O-glucosiole	626.67	49.28	0.62
	MOL000442	1, 7-Dihydroxy-3, 9-dimethoxy pterocarpene	314.31	39.05	0.48
	MOL000098	Quercetin	302.25	46.43	0.28

Fangfeng (FF)	MOL000011	(2R, 3R)-3-(4-Hydroxy-3-methoxy-phenyl)-5-methoxy-2-methylol-2, 3-dihydropyrano[5, 6-h][1,4] benzodioxin-9-one	386.38	68.83	0.66
	MOL011730	11-Hydroxy-sec-o-beta-d-glucosylhamaudol_qt	292.31	50.24	0.27
	MOL011732	Anomalin	426.5	59.65	0.66
	MOL011737	Divaricatacid	320.32	87	0.32
	MOL011740	Divaricatol	334.35	31.65	0.38
	MOL001941	Ammidin	270.3	34.55	0.22
	MOL011747	Ledebouriellol	374.42	32.05	0.51
	MOL011749	Phelloptorin	300.33	43.39	0.28
	MOL011753	5-O-Methylvisamminol	290.34	37.99	0.25
	MOL002644	Phellopterin	300.33	40.19	0.28
	MOL000359	Sitosterol	414.79	36.91	0.75
	MOL000173	Wogonin	284.28	30.68	0.23
	MOL000358	Beta-sitosterol	414.79	36.91	0.75
	MOL001494	Mandenol	308.56	42	0.19
	MOL001942	Isoimperatorin	270.3	45.46	0.23
	MOL003588	Prangenidin	270.3	36.31	0.22
	MOL007514	Methyl icosa-11,14-dienoate	322.59	39.67	0.23
	MOL013077	Decursin	328.39	39.27	0.38

Baizhu (BZ)	MOL000020	12-Senecioyl-2E, 8E, 10E-atractylentriol	312.39	62.4	0.22
	MOL000021	14-Acetyl-12-senecioyl-2E, 8E, 10E-atractylentriol	355.44	60.31	0.31
	MOL000022	14-Acetyl-12-senecioyl-2E, 8Z, 10E-atractylentriol	356.45	63.37	0.3
	MOL000028	*α*-Amyrin	426.8	39.51	0.76
	MOL000033	(3S, 8S, 9S, 10R, 13R, 14S, 17R)-10, 13-Dimethyl-17-[(2R, 5S)-5-propan-2-yloctan-2-yl]-2, 3, 4, 7, 8, 9, 11, 12, 14, 15, 16, 17-dodecahydro-1H-cyclopenta[a] phenanthren-3-ol	428.82	36.23	0.78
	MOL000049	3*β*-Acetoxyatractylone	274.39	54.07	0.22
	MOL000072	8*β*-Ethoxy atractylenolide III	276.41	35.95	0.21

**Table 2:** Top 5 high degree active ingredients in YPFP-Target-Allergy network.

No	Molecule id	Molecule name (active ingredients)	Degree
1	MOL000098	Quercetin	41
2	MOL000422	Kaempferol	21
3	MOL000173	Wogonin	17
4	MOL000392	Formononetin	14
5	MOL000378	7-O-Methylisomucronulatol	11

**Table 3:** Molecular docking parameters and results of key ingredients binding to hub targets.

Key ingredients	Target	Vina score	Cavity size	Center			Size		
				*x*	*y*	*z*	*x*	*y*	*z*
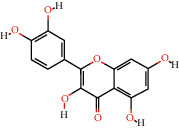 Quercetin	PTGS2	−9.4	4392	13.702	49.228	64.724	21	30	29
	TNF	−8.9	10384	29.389	−21.424	41.646	35	35	35
	IL6	−9.1	2422	−28.73	32.674	41.7	27	21	21
	JUN	−8	1710	−2.548	−5.371	20.862	21	21	32
	IL1B	−6.9	145	−15.895	9.344	−14.260	21	21	21
	CCL2	−7.3	195	−3.977	−17.987	4.759	21	21	21
	IFNG	−7	932	7.332	35.436	22.884	31	21	35
	IL4	−6.9	70	9.769	34.813	−0.368	21	21	21
	IL10	−6.6	73	13.752	61.149	41.144	21	21	21
	CXCL8	−6.2	92	18.369	−13.86	−17.202	21	21	21

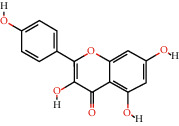 Kaempferol	PTGS2	−9.5	5179	11.893	52.644	16.694	33	21	29
	TNF	−9	10384	29.389	−21.424	41.646	35	35	35
	IL6	−8.8	2422	−28.73	32.674	41.7	27	21	21
	JUN	−7.9	1710	−2.548	−5.371	20.862	21	21	32
	IL1B	−6.6	154	−22.700	21.788	−13.997	21	21	21
	CCL2	−7.1	195	−3.977	−17.987	4.759	21	21	21
	IFNG	−7.1	932	7.332	35.436	22.884	31	21	35
	IL4	−6.4	70	9.769	34.813	−0.368	21	21	21
	IL10	−6.7	162	42.705	34.512	43.604	21	21	21
	CXCL8	−6.2	114	17.866	−9.064	−0.582	21	21	21

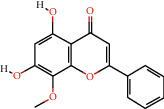 Wogonin	PTGS2	−9.1	5179	11.893	52.644	16.694	33	26	29
	TNF	−9	10384	29.389	−21.424	41.646	35	35	35
	IL6	−8.3	2422	−28.73	32.674	41.7	27	20	20
	JUN	−8.4	1710	−2.548	−5.371	20.862	20	26	32
	IL1B	−6.4	154	−22.700	21.788	−13.997	20	20	20
	CCL2	−6.4	195	−3.977	−17.987	4.759	20	20	20
	IFNG	−6.8	932	7.332	35.436	22.884	31	20	35
	IL4	−6.2	397	30.6	32.102	12.819	20	20	20
	IL10	−7.1	262	5.253	57.499	35.389	20	20	20
	CXCL8	−6.3	114	17.866	−9.064	−0.582	20	20	20

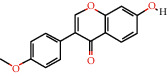 Formononetin	PTGS2	−9.3	5179	11.893	52.644	16.694	33	21	29
	TNF	−8.2	703	32.132	−11.15	−7.15	21	21	21
	IL6	−8	608	−58.308	−3.426	17.841	21	21	21
	JUN	−7.2	1710	−2.548	−5.371	20.862	21	21	32
	IL1B	−6.5	111	−18.334	4.585	5.997	21	21	21
	CCL2	−6.9	195	−3.977	−17.987	4.759	21	21	21
	IFNG	−7.2	932	7.332	35.436	22.884	31	21	35
	IL4	−5.8	70	9.769	34.813	−0.368	21	21	21
	IL10	−6.9	262	5.253	57.499	35.389	21	21	21
	CXCL8	−6.6	114	17.866	−9.064	−0.582	21	21	21

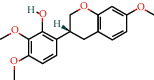 7-O-Methylisomucronulatol	PTGS2	−8.5	5179	11.893	52.644	16.694	33	21	29
	TNF	−8.1	703	32.132	−11.15	−7.15	21	21	21
	IL6	−8.2	2422	−28.73	32.674	41.7	27	21	21
	JUN	−7.2	1710	−2.548	−5.371	20.862	21	21	32
	IL1B	−6.2	145	−15.895	9.344	14.260	21	21	21
	CCL2	−6.1	195	−3.977	−17.987	4.759	21	21	21
	IFNG	−6.5	3791	−2.13	29.796	34.129	21	35	21
	IL4	−5.6	70	9.769	34.813	−0.368	21	21	21
	IL10	−6.9	162	42.705	34.512	43.604	21	21	21
	CXCL8	−5.6	114	17.866	−9.064	−0.582	21	21	21

## Data Availability

The data used to support the findings of this study are available from the corresponding author upon request.

## Supplementary Materials

Supplementary Materials Supplementary Table S1: YPFP-related target genes obtained by TCMSP target gene prediction and UniProt gene name transformation. Supplementary Table S2: target genes corresponding to 5 keywords of “atopic dermatitis,” “atopic eczema,” “asthma,” “allergic rhinitis” and “food allergy.” Supplementary Table S3: node degree of each protein in PPI network. Supplementary Table S4: top 10 in the PPI network ranked by the MCC method. Supplementary Table S5: PDB IDs and references of key proteins.
