# Supplementary material for: Network Pharmacology and Molecular Docking Study of Yupingfeng Powder in the Treatment of Allergic Diseases
Source: Evid Based Complement Alternat Med. 2022 Jul 9;2022:1323744. doi: 10.1155/2022/1323744 (PMC9288288; doi:10.1155/2022/1323744)
Supplement: Supplementary Materials — Supplementary Table S1: YPFP-related target genes obtained by TCMSP target gene prediction and UniProt gene name transformation. Supplementary Table S2: target genes corresponding to 5 keywords of “atopic dermatitis,” “atopic eczema,” “asthma,” “allergic rhinitis” and “food allergy.” Supplementary Table S3: node degree of each protein in PPI network. Supplementary Table S4: top 10 in the PPI network ranked by the MCC method. Supplementary Table S5: PDB IDs and references of key proteins. [file 1323744.f1.zip › Supplementary Table S1.pdf]

Supplementary Table S1

YPFP-related target genes obtained by TCMSP target gene prediction and UniProt gene name transformation.

	**Target Name**	**UniProtKB AC/ID**	**Gene Name**
19 common elements in "BZ", "FF" and "HQ":	Acetylcholinesterase	ACES_HUMAN	ACHE
	Alpha-1A adrenergic receptor	ADA1A_HUMAN	ADRA1A
	Androgen receptor	ANDR_HUMAN	AR
	Beta-2 adrenergic receptor	ADRB2_HUMAN	ADRB2
	Dipeptidyl peptidase IV	DPP4_HUMAN	DPP4
	Gamma-aminobutyric acid receptor subunit alpha-1	GBRA1_HUMAN	GABRA1
	Muscarinic acetylcholine receptor M1	ACM1_HUMAN	CHRM1
	Muscarinic acetylcholine receptor M2	ACM2_HUMAN	CHRM2
	Muscarinic acetylcholine receptor M3	ACM3_HUMAN	CHRM3
	Mu-type opioid receptor	OPRM_HUMAN	OPRM1
	Neuronal acetylcholine receptor protein, alpha-7 chain	ACHA7_HUMAN	CHRNA7
	Nitric-oxide synthase, endothelial	NOS3_HUMAN	NOS3
	Nuclear receptor coactivator 1	NCOA1_HUMAN	NCOA1
	Nuclear receptor coactivator 2	NCOA2_HUMAN	NCOA2
	Progesterone receptor	PRGR_HUMAN	PGR
	Prostaglandin G/H synthase 2	PGH2_HUMAN	PTGS2
	Retinoic acid receptor RXR-alpha	RXRA_HUMAN	RXRA
	Sodium channel protein type 5 subunit alpha	SCN5A_HUMAN	SCN5A
	Thrombin	THRB_HUMAN	F2
57 common elements in "FF" and "HQ":	Alpha-1B adrenergic receptor	ADA1B_HUMAN	ADRA1B
	Coagulation factor VII	FA7_HUMAN	F7
	Calcium-activated potassium channel subunit alpha 1	KCMA1_HUMAN	KCNMA1
	Apoptosis regulator BAX	BAX_HUMAN	BAX
	Apoptosis regulator Bcl-2	BCL2_HUMAN	BCL2
		CALM1_HUMAN	CALM1
	Calmodulin	CALM2_HUMAN	CALM2
		CALM3_HUMAN	CALM3
	Caspase-3	CASP3_HUMAN	CASP3
	Caspase-9	CASP9_HUMAN	CASP9
	Cell division protein kinase 2	CDK2_HUMAN	CDK2
	CGMP-inhibited 3',5'-cyclic phosphodiesterase A	PDE3A_HUMAN	PDE3A
	Coagulation factor Xa	FA10_HUMAN	F10
	Cyclin-A2	CCNA2_HUMAN	CCNA2
	Amine oxidase [flavin-containing] B	AOFB_HUMAN	MAOB
	DNA topoisomerase II	TOP2B_HUMAN	TOP2B
	Dopamine D1 receptor	DRD1_HUMAN	DRD1

Estrogen receptor	ESR1_HUMAN	ESR1
Estrogen receptor beta	ESR2_HUMAN	ESR2
Glycogen synthase kinase-3 beta	GSK3B_HUMAN	GSK3B
Heat shock protein HSP 90	HS90A_HUMAN<br>HS902_HUMAN	HSP90AA1<br>HSP90AA2P
mRNA of PKA Catalytic Subunit C-alpha	KAPCA_HUMAN	PRKACA
mRNA of Protein-tyrosine phosphatase, non-receptor type 1	PTN1_HUMAN	PTPN1
Nitric oxide synthase, inducible	NOS2_HUMAN	NOS2
Mitogen-activated protein kinase 14	MK14_HUMAN	MAPK14
Peroxisome proliferator activated receptor gamma	PPARG_HUMAN	PPARG
Transcription factor p65	TF65_HUMAN	RELA
RAC-alpha serine/threonine-protein kinase	AKT1_HUMAN	AKT1
G1/S-specific cyclin-D1	CCND1_HUMAN	CCND1
Potassium voltage-gated channel subfamily H member 2	KCNH2_HUMAN	KCNH2
Cyclin-dependent kinase inhibitor 1	CDN1A_HUMAN	CDKN1A
Eukaryotic translation initiation factor 6	IF6_HUMAN	EIF6
Tumor necrosis factor	TNFA_HUMAN	TNF
Interleukin-6	IL6_HUMAN	IL6
Activator of 90 kDa heat shock protein ATPase homolog 1	AHSA1_HUMAN	AHSA1
Prostaglandin G/H synthase 1	PGH1_HUMAN	PTGS1
Cellular tumor antigen p53	P53_HUMAN	TP53
Interstitial collagenase	MMP1_HUMAN	MMP1
C-C motif chemokine 2	CCL2_HUMAN	CCL2
Prostaglandin E2 receptor EP3 subtype	PE2R3_HUMAN	PTGER3
Interleukin-8	IL8_HUMAN	CXCL8
Gamma-aminobutyric-acid receptor alpha-2 subunit	GBRA2_HUMAN	GABRA2
Muscarinic acetylcholine receptor M4	ACM4_HUMAN	CHRM4
5-hydroxytryptamine 2A receptor	5HT2A_HUMAN	HTR2A
Gamma-aminobutyric-acid receptor alpha-5 subunit	GBRA5_HUMAN	GABRA5
Gamma-aminobutyric-acid receptor alpha-3 subunit	GBRA3_HUMAN	GABRA3
Sodium-dependent serotonin transporter	SC6A4_HUMAN	SLC6A4
Proto-oncogene serine/threonine-protein kinase Pim-1	PIM1_HUMAN	PIM1
Caspase-8	CASP8_HUMAN	CASP8
Protein kinase C alpha type	KPCA_HUMAN	PRKCA
Transforming growth factor beta-1	TGFB1_HUMAN	TGFB1
Serum paraoxonase/arylesterase 1	PON1_HUMAN	PON1

	Serine/threonine-protein kinase Chk1	CHK1_HUMAN	CHEK1
	Transcription factor AP-1	JUN_HUMAN	JUN
	Trypsin-1	TRY1_HUMAN	PRSS1
	Vascular endothelial growth factor receptor 2	VGFR2_HUMAN	KDR
9 elements included exclusively in "FF":	Mineralocorticoid receptor	MCR_HUMAN	NR3C2
	Bcl-2-binding component 3	BBC3_HUMAN	BBC3
	Telomerase protein component 1	TEP1_HUMAN	TEP1
	Protein kinase C delta type	KPCD_HUMAN	PRKCD
	Fibronectin	FINC_HUMAN	FN1
	Induced myeloid leukemia cell differentiation protein Mcl-1	MCL1_HUMAN	MCL1
	Neuronal acetylcholine receptor subunit alpha-2	ACHA2_HUMAN	CHRNA2
	Microtubule-associated protein 2	MTAP2_HUMAN	MAP2
	Carbonic anhydrase II	CAH2_HUMAN	CA2
136 elements included exclusively in "HQ":	Xanthine dehydrogenase/oxidase	XDH_HUMAN	XDH
	Vascular endothelial growth factor A	VEGFA_HUMAN	VEGFA
	Va136 scular cell adhesion protein 1	VCAM1_HUMAN	VCAM1
	Urokinase-type plasminogen activator	UROK_HUMAN	PLAU
	Type I iodothyronine deiodinase	IOD1_HUMAN	DIO1
	Transcription factor E2F2	E2F2_HUMAN	E2F2
	Transcription factor E2F1	E2F1_HUMAN	E2F1
	Tissue-type plasminogen activator	TPA_HUMAN	PLAT
	Tissue factor	TF_HUMAN	F3
	Thrombomodulin	TRBM_HUMAN	THBD
	Superoxide dismutase [Cu-Zn]	SODC_HUMAN	SOD1
	Stromelysin-1	MMP3_HUMAN	MMP3
	Solute carrier family 2, facilitated glucose transporter member 4	GLUT4_HUMAN	SLC2A4
	Sodium-dependent noradrenaline transporter	SC6A2_HUMAN	SLC6A2
	Sodium-dependent dopamine transporter	SC6A3_HUMAN	SLC6A3
	Signal transducer and activator of transcription 1-alpha/beta	STAT1_HUMAN	STAT1
	Serine/threonine-protein phosphatase 2B catalytic subunit alpha isoform	PP2BA_HUMAN	PPP3CA
	Serine/threonine-protein kinase Chk2	CHK2_HUMAN	CHEK2
	Runt-related transcription factor 2	RUNX2_HUMAN	RUNX2
	Retinoic acid receptor RXR-beta	RXRB_HUMAN	RXRB
	Retinoblastoma-associated protein	RB_HUMAN	RB1
	Receptor tyrosine-protein kinase erbB-3	ERBB3_HUMAN	ERBB3
	Receptor tyrosine-protein kinase erbB-2	ERBB2_HUMAN	ERBB2
	Ras GTPase-activating protein 1	RASA1_HUMAN	RASA1
	Ras association domain-containing protein 1	RASF1_HUMAN	RASSF1
	RAF proto-oncogene serine/threonine-protein kinase	RAF1_HUMAN	RAF1

Puromycin-sensitive aminopeptidase	PSA_HUMAN	NPEPPS
Proto-oncogene c-Fos	FOS_HUMAN	FOS
Protein kinase C beta type	KPCB_HUMAN	PRKCB
Protein CBFA2T1	MTG8_HUMAN	RUNX1T1
Prostatic acid phosphatase	PPAP_HUMAN	ACP3
Pro-epidermal growth factor	EGF_HUMAN	EGF
Procollagen C-endopeptidase enhancer 1	PCOC1_HUMAN	PCOLCE
Probable E3 ubiquitin-protein ligase HERC5	HERC5_HUMAN	HERC5
Poly [ADP-ribose] polymerase 1	PARP1_HUMAN	PARP1
Plasminogen activator inhibitor 1	PAI1_HUMAN	SERPINE1
Phosphatidylinositol-3,4,5-trisphosphate 3-phosphatase and dual-specificity protein phosphatase PTEN	PTEN_HUMAN	PTEN
Peroxisome proliferator-activated receptor delta	PPARD_HUMAN	PPARD
Peroxisome proliferator-activated receptor alpha	PPARA_HUMAN	PPARA
Oxidized low-density lipoprotein receptor 1	OLR1_HUMAN	OLR1
Osteopontin	OSTP_HUMAN	SPP1
Ornithine decarboxylase	DCOR_HUMAN	ODC1
Nuclear receptor subfamily 1 group I member 3	NR1I3_HUMAN	NR1I3
Nuclear receptor subfamily 1 group I member 2	NR1I2_HUMAN	NR1I2
Nuclear factor erythroid 2-related factor 2	NF2L2_HUMAN	NFE2L2
NF-kappa-B inhibitor alpha	IKBA_HUMAN	NFKBIA
Neutrophil cytosol factor 1	NCF1_HUMAN	NCF1
NADPH--cytochrome P450 reductase	NCPR_HUMAN	POR
NADH-ubiquinone oxidoreductase chain 6	NU6M_HUMAN	MT-ND6
NAD-dependent deacetylase sirtuin-1	SIR1_HUMAN	SIRT1
NAD(P)H dehydrogenase [quinone] 1	NQO1_HUMAN	NQO1
Myeloperoxidase	PERM_HUMAN	MPO
Myc proto-oncogene protein	MYC_HUMAN	MYC
Muscarinic acetylcholine receptor M5	ACM5_HUMAN	CHRM5
Mitogen-activated protein kinase 8	MK08_HUMAN	MAPK8
Mitogen-activated protein kinase 1	MK01_HUMAN	MAPK1
Matrix metalloproteinase-9	MMP9_HUMAN	MMP9
Maltase-glucoamylase, intestinal	MGA_HUMAN	MGAM
Lysozyme	LYSC_HUMAN	LYZ
Interleukin-4	IL4_HUMAN	IL4
Interleukin-2	IL2_HUMAN	IL2
Interleukin-10	IL10_HUMAN	IL10
Interleukin-1 beta	IL1B_HUMAN	IL1B
Interleukin-1 alpha	IL1A_HUMAN	IL1A
Interferon regulatory factor 1	IRF1_HUMAN	IRF1
Interferon gamma	IFNG_HUMAN	IFNG
Intercellular adhesion molecule 1	ICAM1_HUMAN	ICAM1
Insulin-like growth factor-binding protein 3	IBP3_HUMAN	IGFBP3

Insulin-like growth factor II	IGF2_HUMAN	IGF2
Insulin receptor	INSR_HUMAN	INSR
Inhibitor of nuclear factor kappa-B kinase subunit beta	IKKB_HUMAN	IKBKB
Inhibitor of nuclear factor kappa-B kinase subunit alpha	IKKA_HUMAN	CHUK
Ig gamma-1 chain C region	IGHG1_HUMAN	IGHG1
Hypoxia-inducible factor 1-alpha	HIF1A_HUMAN	HIF1A
Hyaluronan synthase 2	HYAS2_HUMAN	HAS2
Homeobox protein Nkx-3.1	NKX31_HUMAN	NKX3-1
Hexokinase-2	HXK2_HUMAN	HK2
Hepatocyte growth factor receptor	MET_HUMAN	MET
Heme oxygenase 1	HMOX1_HUMAN	HMOX1
Heat shock protein beta-1	HSPB1_HUMAN	HSPB1
Heat shock factor protein 1	HSF1_HUMAN	HSF1
Glycogen phosphorylase, muscle form	PYGM_HUMAN	PYGM
Glutathione S-transferase P	GSTP1_HUMAN	GSTP1
Glutathione S-transferase Mu 2	GSTM2_HUMAN	GSTM2
Glutathione S-transferase Mu 1	GSTM1_HUMAN	GSTM1
Glutamate receptor 2	GRIA2_HUMAN	GRIA2
Gap junction alpha-1 protein	CXA1_HUMAN	GJA1
Gamma-aminobutyric-acid receptor subunit alpha-6	GBRA6_HUMAN	GABRA6
G2/mitotic-specific cyclin-B1	CCNB1_HUMAN	CCNB1
ETS domain-containing protein Elk-1	ELK1_HUMAN	ELK1
Estrogen sulfotransferase	ST1E1_HUMAN	SULT1E1
E-selectin	LYAM2_HUMAN	SELE
Epidermal growth factor receptor	EGFR_HUMAN	EGFR
Dual oxidase 2	DUOX2_HUMAN	DUOX2
DNA topoisomerase 2-alpha	TOP2A_HUMAN	TOP2A
DNA topoisomerase 1	TOP1_HUMAN	TOP1
Delta-type opioid receptor	OPRD_HUMAN	OPRD1
DDB1- and CUL4-associated factor 5	DCAF5_HUMAN	DCAF5
Cytochrome P450 3A4	CP3A4_HUMAN	CYP3A4
Cytochrome P450 1B1	CP1B1_HUMAN	CYP1B1
Cytochrome P450 1A2	CP1A2_HUMAN	CYP1A2
Cytochrome P450 1A1	CP1A1_HUMAN	CYP1A1
Cyclin-dependent kinase inhibitor 2A, isoforms 1/2/3	CDN2A_HUMAN	CDKN2A
C-X-C motif chemokine 2	CXCL2_HUMAN	CXCL2
C-X-C motif chemokine 11	CXL11_HUMAN	CXCL11
C-X-C motif chemokine 10	CXL10_HUMAN	CXCL10
C-reactive protein	CRP_HUMAN	CRP

	Collagen alpha-1(III) chain	CO3A1_HUMAN	COL3A1
	Collagen alpha-1(I) chain	CO1A1_HUMAN	COL1A1
	Claudin-4	CLD4_HUMAN	CLDN4
	Cell division control protein 2 homolog	CDC42_HUMAN	CDC42
	CD40 ligand	CD40L_HUMAN	CD40LG
	Caveolin-1	CAV1_HUMAN	CAV1
	Cathepsin D	CATD_HUMAN	CTSD
	cAMP-dependent protein kinase inhibitor alpha	IPKA_HUMAN	PKIA
	Beta-lactamase	DPEP1_HUMAN	DPEP1
	Beta-1 adrenergic receptor	ADRB1_HUMAN	ADRB1
	Bcl-2-like protein 1	B2CL1_HUMAN	BCL2L1
	Baculoviral IAP repeat-containing protein 5	BIRC5_HUMAN	BIRC5
	ATP synthase subunit beta, mitochondrial	ATPB_HUMAN	ATP5F1B
	Aryl hydrocarbon receptor	AHR_HUMAN	AHR
	Arachidonate 5-lipoxygenase	LOX5_HUMAN	ALOX5
	Antileukoproteinase	SLPI_HUMAN	SLPI
	Alpha-2C adrenergic receptor	ADA2C_HUMAN	ADRA2C
	Alpha-1D adrenergic receptor	ADA1D_HUMAN	ADRA1D
	Aldose reductase	AK1BA_HUMAN	AKR1B10
	Aldo-keto reductase family 1 member C3	AK1C3_HUMAN	AKR1C3
	Alcohol dehydrogenase 1C	ADH1G_HUMAN	ADH1C
	Alcohol dehydrogenase 1B	ADH1B_HUMAN	ADH1B
	Acetyl-CoA carboxylase 1	ACACA_HUMAN	ACACA
	78 kDa glucose-regulated protein	BIP_HUMAN	HSPA5
	72 kDa type IV collagenase	MMP2_HUMAN	MMP2
	5-hydroxytryptamine receptor 3A	5HT3A_HUMAN	HTR3A
	3 beta-hydroxysteroid dehydrogenase/Delta 5-->4-isomerase type 2	3BHS2_HUMAN	HSD3B2
	3 beta-hydroxysteroid dehydrogenase/Delta 5-->4-isomerase type 1	3BHS1_HUMAN	HSD3B1
	26S proteasome non-ATPase regulatory subunit 3	PSMD3_HUMAN	PSMD3
