# Supplementary material for: Network Pharmacology and Molecular Docking Study of Yupingfeng Powder in the Treatment of Allergic Diseases
Source: Evid Based Complement Alternat Med. 2022 Jul 9;2022:1323744. doi: 10.1155/2022/1323744 (PMC9288288; doi:10.1155/2022/1323744)
Supplement: Supplementary Materials — Supplementary Table S1: YPFP-related target genes obtained by TCMSP target gene prediction and UniProt gene name transformation. Supplementary Table S2: target genes corresponding to 5 keywords of “atopic dermatitis,” “atopic eczema,” “asthma,” “allergic rhinitis” and “food allergy.” Supplementary Table S3: node degree of each protein in PPI network. Supplementary Table S4: top 10 in the PPI network ranked by the MCC method. Supplementary Table S5: PDB IDs and references of key proteins. [file 1323744.f1.zip › Supplementary Table S2.pdf]

Supplementary Table S2

Target genes corresponding to 5 keywords of "atopic dermatitis", "atopic eczema", "asthma", "allergic rhinitis" and "food allergy".

atopic dermatitis	atopic eczema	asthma	allergic rhinitis	food allergy
FLG	FLG	ALRH	IL13	IGHE
ATOD3	ATOD3	AS1	IL4R	IL13
ATOD6	ATOD6	ASRT1	IL4	FLG
ATOD5	ATOD5	ASRT2	IL5	IL4
ATOD8	ATOD1	ASRT5	IGHE	TNF
ATOD7	ATOD8	ASRT7	RNASE3	FOXP3
ATOD9	ATOD7	BHR1	FLG	IL10
ATOD1	ATOD9	GP39	MS4A2	IL5
CARD11	IL4R	GPR154	CCL11	SPINK5
MIR155	MIR155	GPRA	IFNG	CSN1S1
FLG-AS1	MS4A2	IMD88	IL10	DSG1
SOCS3	CARD11	IRAKM	HRH1	IGES
IL13	IL13	MRT51	TNF	RNASE3
IL4	IL4	PAFAD	CXCL8	IL4R
IL4R	SPINK5	PAFAH	ALRH	IFNG
IGHE	IGHE	PGR14	CCL5	ALB
IFNG	IFNG	SCYA11	IL17A	ENPP3
MS4A2	FLG-AS1	TBET	EPX	STAT6
IL5	WAS	TNFA	TSLP	INS
TNF	IL5	UGRP1	ICAM1	GHRL
SPINK5	SOCS3	VRR1	IL6	CTLA4
IL10	STAT1	YKL40	FOXJ1	HLA-DQB1
RNASE3	TNF	A2M	CCR3	CETP
CCL11	IL21R	AAA1	IL18	TLR4
NOD2	RNASE3	ABCB1	FOXP3	JAK1
IL31	PLA2G7	ABCC1	GATA3	DSP
TSLP	TSLP	ABI3BP	MIR155	DSG1-AS1
CCL17	CCL11	ABL1	IL33	LEP
FOXP3	FOXP3	ABO	PLA2G7	SCP2
CCL22	CTLA4	ACAA1	IL9	CCL11
CXCL8	STAT3	ACE	IGES	CXCL8
IL31RA	IL10	ACE2	NGF	HLA-DRB1
IL2	IL31	ACKR1	VCAM1	PVALB
IL2RA	PGM3	ACO1	CCL17	MS4A2
IL18	DOCK8	ACP1	ALOX5	IL6
CCL5	CMA1	ACVR1	IL21R	IL17A
IL6	IGES	ACVRL1	IL1B	AOC1
MSMO1	CCL17	ADA	MPO	TLR2
CTLA4	CCL5	ADAM12	CSF2	CSN3
TLR2	TLR2	ADAM17	IL16	EPX

PGM3	IL18	ADAM23	CCL2	LALBA
IL17A	IL2RA	ADAM33	ATOD1	PGM3
CCR4	IL2	ADAM8	ATOD6	NPY
ZNF750	CXCL8	ADAMTS9	ATOD5	HRH1
IL1B	PHF11	ADAMTSL1	IL2	HLA-B
GATA3	IL17A	ADCY10	ATOD3	EOE2
JAK1	TLR4	ADCY9	VIP	MIR155
ICAM1	FCER2	ADCYAP1	FCER2	SLC27A4
STAT3	IL6	ADCYAP1R1	ATOD7	CCK
HLA-DRB1	KLK7	ADH5	ATOD8	IL1B
KLK7	CCL22	ADIPOQ	ATOD9	IL6ST
IGES	NOD2	ADM	TLR4	EOE1
DSG1	GATA3	ADORA1	CCL24	G6PD
IL2RB	STAT6	ADORA2A	FCER1A	FCER2
STAT1	IL31RA	ADORA2B	PRG2	TSLP
IL33	CCR4	ADORA3	HLA-DRB1	GCG
CCL27	IL1B	ADRA1A	ALB	TPT1
TLR4	CCL27	ADRA1B	IL3	CCR6
CSF2	IL33	ADRB1	SELE	ADRB2
DEFB4A	CAMP	ADRB2	IL2RA	HLA-DQA1
IL16	DEFB4A	ADRB3	NR3C1	CARMIL2
FCER2	CSF2	AFF4	TLR2	BRCA2
CXCR3	ICAM1	AGER	KNG1	POMC
LORICRIN	IL7R	AGL	CD14	CD79A
PHF11	IL16	AGT	ASRT4	CD4
CDSN	CD14	AGTR1	TPT1	CRP
SELE	IL6R	AHR	CTLA4	TGFB1
IL1A	JAK1	AICDA	CCR4	RBM8A
CCR3	TLR9	AIF1	CYSLTR1	IL33
IL6R	CCL18	AIMP1	HAVCR1	PLCG2
IL9	CCR6	AKR1B1	PTGDR2	LEPR
IL21R	CSN1S1	AKR1C3	CCL22	PDCL
CCL2	LORICRIN	AKT1	IL31	IL2
STAT6	CCR3	ALAD	CLC	GAST
CMA1	EPX	ALB	CCL3	IL9
NGF	CDSN	ALDH2	SDAD1	CYP3A4
PLA2G7	IL9	ALG9	CXCR3	CD63
IVL	IL1A	ALMS1	SPINK5	IL2RB
SELL	CXCR3	ALOX12	HLA-DQB1	MPO
CCL18	TMEM79	ALOX15	CCL26	IL3
CCR6	HAVCR1	ALOX5	STAT6	ATOD1
HLA-DQB1	CCL2	ALOX5AP	CALCA	IL18
CXCL10	PRG2	ALPP	IL1A	ATOD6
FAF2	DEFB103B	ANGPT1	HLA-B	ATOD5

TMEM79	CRLF2	ANGPT2	DNAH5	SPTBN1
TGM3	ZNF341	ANK1	SCGB1A1	NAT2
CSN1S1	CSTA	ANKEF1	IL1RL1	ICAM1
PRG2	NGF	ANO3	F2RL1	SYK
EPX	IVL	ANPEP	TLR9	ATOD3
VCAM1	STAT5B	ANXA1	SYK	SOX6
TACR1	HLA-DRB1	ANXA2	TAC1	CD19
BDNF	NPSR1	ANXA5	TGFB1	ATOD7
HRH1	CCL26	AOAH	SELL	ATOD8
CAMP	TACR1	AOC1	TBXA2R	ATOD9
CCL26	SELL	APC	SOCS3	MIR126
FOXN1	HRH1	APOA1	ELANE	GATA3
CD8A	IL1RL1	APOE	ADRB2	CYP2C9
LTA	LTA	AQP2	CD4	GSTM1
CXCL9	TPMT	AQP5	HLA-G	CCR9
S100A7	DOP1A	AR	SFTPD	PTGS2
CD4	TGM3	AREG	CCL7	IAPP
ITGAM	S100A7	ARG1	ADAM33	ESR1
ZNF341	CXCL10	ARG2	BDNF	DOCK8
HAVCR1	IL10RA	ARL6	PLCG2	MIR148A
PDE4A	CXCL9	ARRB2	CD40LG	OXA1L
TLR9	TNFRSF8	ARSL	CCR6	CD14
CYP4F22	IL2RB	ARVCF	ICOSLG	HNMT
NPSR1	CD28	ASIC4-AS1	ENPP3	TP53
HRH4	BDNF	ASOBS	DEFB4A	OPRM1
DEFB103B	TIMP1	ASRT3	CD86	CSN2
PTGDR2	HLA-DQB1	ASRT4	CCL13	MTOR
SYK	LALBA	ASRT6	TLR3	PLA2G7
IL12RB1	SELE	ASRT8	CD40	ALOX5
IL7R	ITGAM	ASTN2	CX3CR1	ICOSLG
FCGR3A	PPBP	ATF3	CD69	PTPN22
IL1RL1	PTGDR2	ATF6	CXCL10	IL2RA
IL7	CD69	ATF6B	CFTR	PYY
CD69	SELP	ATG3	CMA1	LGALS3
CCL20	IL12RB1	ATG5	ITGAM	NOD2
TIMP1	CCL7	ATOD1	MBL2	ATP12A
TMPO	CD8A	ATOD3	NPY	ATP4A
MIF	VCAM1	ATOD5	CD79A	ACE
DOCK8	IL3	ATOD6	S100A7	CD40LG
SELP	CCL20	ATOD7	HNMT	CCL22
LALBA	PDE4A	ATOD8	AOC1	ADIPOQ
CRLF2	FOXN1	ATOD9	ACE	CD69
F2RL1	CX3CR1	ATP12A	IL1RN	PDC
CHI3L1	HRH4	ATP2A2	CLEC7A	MTHFR

TNFRSF8	KRT1	ATP2A3	ITGAL	COPS7A
CYSLTR1	CYP4F22	ATP4A	TAP1	KNG1
PSIP1	CYSLTR1	ATRIP	IL17F	CCR3
CCL7	FAF2	ATRIP-TREX1	TNFRSF8	PPARG
CD40	VIP	AXL	CSN1S1	TGFBR2
CX3CR1	FCGR3A	B3GAT1	TAP2	HLA-A
IRAK3	TMPO	BAX	IL12RB1	GHITM
PPBP	CD4	BBIP1	TACR1	ADA
PTGDR	F2RL1	BBS1	ITGB2	EMSY
IL10RA	KITLG	BBS10	CDSN	STAT3
CSTA	HLA-DQA1	BBS12	CXCR1	FCER1A
CARMIL2	CCL4	BBS2	PTGDR	CYP2E1
DSP	PSIP1	BBS4	CYP3A4	CCL17
CCL4	MIF	BBS5	NPSR1	CFTR
TPMT	TAC1	BBS7	SFTPA1	PTGDR2
VIP	LGALS3	BBS9	KITLG	MIR21
KITLG	CCL13	BCDIN3D	POSTN	KRAS
TAC1	LRRC32	BCL11B	CCL4	TGM2
NTF3	CD40	BCL2	ITK	CCL26
CCL13	CCL1	BCL2A1	NOD2	UGT1A1
CCL1	NTF3	BCR	PTGS1	PEDS1
HLA-DQA1	COL6A5	BDKRB1	CCL20	MT-TL1
KCNJ11	CFTR	BDKRB2	CCL27	IFNGR1
BTD	DEFB103A	BDNF	IL1R1	HCRT
IL17D	CSN3	BECN1	GSTP1	HLA-DPB1
DOP1A	IL17D	BEST1	HLA-C	CALCA
CD14	KCNJ11	BGLAP	NTF3	CRH
CTSE	CHI3L1	BIRC3	MUC5AC	CCL5
IL3	CTSE	BIRC5	NAT2	PTGS1
SERPINB4	CD86	BMP2	TRPV1	VIP
CSN3	SYK	BMP6	EDN1	CYP2D6
HLA-B	SOCS1	BMP7	STAT3	CMA1
CD86	TOM1	BMPR1B	IL15	GAL
LGALS3	PTGDR	BMPR2	HRH4	NOS3
FCGR1A	FCGR1A	BPI	NOS2	CNR1
TXK	IRAK3	BRCA2	POMC	CXCL10
CFTR	TXK	BRD2	SERPINA3	CCL2
CD83	ALOX5	BST2	SELP	IGF1
LRRC32	ICOSLG	BTK	TGFBR1	BDNF
PSMB9	CD83	BTNL2	HLA-A	LACTB
ICOSLG	IL6ST	C11orf71	IL22	MIR152
BRAF	EDA	C19orf12	SIK3	EPO
CCR10	ICAM3	C1orf100	PPBP	CDKN2A
SERPINA3	HLCS	C3	IL7	CCR5

ICAM3	SERPINA3	C3AR1	CCR7	DOP1A
BCL7B	IL7	C4A	CD28	TLR9
ADAM33	SERPINB4	C5	CARMIL2	MGAM
NAT2	SHOC2	C5AR1	HLA-DQA1	KIT
IL22	IFIH1	C5orf46	LTC4S	GPT
MIR142	NCF4	C6orf118	LTA	OXT
KRT1	MIR142	C9orf24	PHF11	ELN
IL1RN	ADRB2	CA10	IL37	HDC
CCR7	KRT16	CACNG2-DT	HP	TGFBR1
CCL3	CARMIL2	CACNG6	TLR7	HLA-G
DEFB103A	IFNGR1	CALB2	CCR5	IL7
CD79A	BRCA2	CALCA	IL18R1	IL1RN
IFIH1	ADAM33	CALR	CCL1	MIF
TGFB1	LBR	CAMP	HLA-DPB1	PRG2
CARD11-AS1	DOCK8-AS1	CANX	CSF1	TPM2
ALOX5	TP63	CARD11	FCGR3A	SLC5A1
KRT16	HNMT	CARMIL2	PDCD1	FASLG
RNASE2	BCL7B	CASP1	PSORS1C1	SELL
IL1R1	RAC1	CASP10	TNFSF11	TPM3
IL12B	BRAF	CASP3	HYDIN	NOS1
HLCS	IL12B	CASP8	HAVCR2	IL15
NACA	DEL18Q	CASR	CD8A	FTO
HNMT	TNFRSF1B	CAT	TIMP1	TPM1
DSC1	SCNN1A	CAV1	TGFBR2	CD40
IL17F	HAVCR2	CCDC28B	PDE4A	CSF2
COL6A5	BTD	CCHCR1	IPO8	GSTP1
MMP9	RNASE2	CCL1	MMP9	HP
EGFR	CCR10	CCL11	PGM3	APC
CALCA	SCGB3A2	CCL13	CCL18	SI
SCNN1A	SCNN1B	CCL15	NFKB1	FABP2
OSMR	HSPA9	CCL17	EMSY	KCNJ11
CD28	NEK9	CCL18	SFTPA2	MIR34A
HLA-A	RRAS2	CCL19	SPEF2	FBN1
GSTM1	SCNN1G	CCL2	CXCL9	GSR
SCNN1B	MORC2	CCL20	TLR8	ERBB2
HSPA9	HLA-G	CCL21	RSPH1	F2
NEK9	DSC1	CCL22	SPAG1	CSTA
RRAS2	TGFB1	CCL24	DNAAF5	SLC6A4
SCNN1G	FLI1	CCL26	CCDC65	APOE
MORC2	FCER1A	CCL27	CFAP298	IL10RA
NLRP3	ELANE	CCL3	DNAAF11	FCGR2A
FCGR3B	NACA	CCL4	MCIDAS	MYLK
CD40LG	CD79A	CCL5	ODAD2	BCL11B
TBX21	PSMB9	CCL7	PI3	CCR4

TGM1	CCL3	CCL8	DNAL1	IL16
IL15	MMP9	CCN2	RSPH4A	CD83
KIT	IL5RA	CCN4	RSPH9	TAC1
PI3	DSG1	CCND1	CSF3	SLC6A3
MMP1	TRAF6	CCR1	TBX21	ACE2
SOCS1	HLA-B	CCR10	EGFR	FCGR3A
GZMB	NFKBIA	CCR2	FCGR1A	DOCK8-AS1
HLA-G	IL21R-AS1	CCR3	CRLF2	VCAM1
CXCL11	ASRT3	CCR4	LRRC32	CD8A
CD1D	ASRT4	CCR5	BGLAP	LTF
HLA-C	ASRT6	CCR5AS	SLC27A4	SOD1
GSTP1	ITGB2	CCR6	COX4I2	GRP
ADRB2	CD40LG	CCR7	IDO1	CCL3
TP63	IRF3	CCR8	CAMP	TPM4
FLG2	CCL24	CCR9	CXCL5	MLXIPL
RMRP	ZNF750	CD14	SCGB3A2	NCF1
NCKAP1L	IDO1	CD160	IL5RA	CCL24
AMBP	CASP8	CD163	IL12B	RBFOX1
IFNGR1	CCR7	CD1D	DNAI2	ABCB1
ITGAX	GZMB	CD200	ZMYND10	MAOA
ICOS	MIR148B	CD209	CCDC40	STAT1
IL6ST	NOD1	CD247	DNAH1	MLN
AK2	IRF2	CD27	CCDC103	B2M
CCL24	GSTM1	CD274	CCDC39	SERPINB7
KRAS	NCF1	CD276	TLR1	CCL7
SLCO2A1	KIF3A	CD28	VEGFA	GZMB
FCER1A	MBTPS2	CD36	DNAAF2	LCT
LBR	ITK	CD38	CRP	TOR1A
DEFB125	AQP3	CD4	IL2RB	LTA
DEFB127	TGFBR1	CD40	PTPN22	MIR148B
DEFB132	ITGAX	CD40LG	LGALS3	TF
DEFB124	MUC7	CD44	SERPINE1	GLP1R
ASAH2B	GBA	CD48	DNAH11	ELANE
LCE1B	HRNR	CD63	TGFB2	CPA3
DEFB128	TBX21	CD69	IL11	PTH
TAP1	PPP1CB	CD79A	RNASE2	MIR122
AHR	BZX	CD80	IL31RA	NOS2
INS	CXCL11	CD83	TRPM8	SELE
ALB	POMC	CD86	ALOX5AP	NLRP3
TOM1	GNA11	CD8A	FASLG	RAC2
NCR2	CD3G	CDH1	NEU1	LCOR
MAP2K1	NR3C1	CDH23	CCNO	SST
LCE3B	MPO	CDH26	DNAI1	CARD11
GBA	OVOL1	CDHR3	OFD1	EDNRA

CSF1	SCGB1A1	CDK2	RPGR	PPIC
HFE	KRT9	CDKN1C	TTC12	CYP1A1
TNFRSF1B	GSTP1	CDON	DNAAF1	F3
LCE5A	TPT1	CDSN	RSPH3	IL1A
LCE3C	IL1R1	CEACAM3	DNAAF3	TPO
SCGB3A2	FADS1	CEACAM6	DRC1	ACP1
CCR5	IL18R1	CEBPA	DNAAF4	MIR145
C4A	TAP1	CEBPB	ODAD3	TAS2R38
CARD14	ALB	CEP19	ODAD1	TCF20
TOR1B	CSF1	CETP	ICAM3	TPMT
IL21	PEPD	CFD	DEFB103B	OTC
ASRT3	POLE	CFH	FCGR2A	TRPV1
ASRT4	DNAJC21	CFHR1	DNAH9	SLCO1B1
ASRT6	IL12RB2	CFL1	CLEC16A	MRGPRX2
IL37	CRH	CFTR	TNFSF4	CSNK2B
UROD	TNFRSF18	CFTR-AS1	LALBA	CCL4
C5	RIT1	CHAT	PTGS2	SERPINA1
DEL18Q	MIR126	CHGA	CYSLTR2	MIR143
HPGD	MIR148A	CHI3L1	IL17D	MIR221
NOD1	MIR152	CHIA	CSN3	MIR146A
CD1C	ASRT8	CHIT1	MRGPRX2	CLMP
HRNR	EOE1	CHML	CXCL2	TREH
MPO	EOE2	CHRM1	PIK3CG	BBS1
TNFSF4	PTPRC	CHRM2	FN1	MIR17
IL26	MUC5AC	CHRM3	LTF	CASP8
GSTT1	MMP1	CHRNA7	CD80	VPS13B
LRP1	FADS2	CHST8	MBP	PTGDS
TGM2	FCER1G	CHUK	CCR8	CYP1A2
ELN	CARD14	CIITA	TNFRSF4	ASXL1
IL12RB2	FBN1	CLC	NOS3	IRF1
C4B	NCF4-AS1	CLCA1	MIF	ALDH2
FCGR2A	TGM5	CLCA4	NOS1	MIR27A
HAVCR2	ICOS	CLCN3	FCER1G	DEL18Q
TAP2	FECH	CLEC16A	GSTT1	CXCR3
NLRP1	TGFB2	CLEC1A	CXCL11	KITLG
EDN1	IRF7	CLEC7A	GAS8	VDR
CCL21	IL22	CLIC2	DNAJB13	MIR30E
PSORS1C1	HMGB1	CLU	ODAD4	SKIV2L
STAT4	NOS2	CMA1	CFAP300	MB
HAO1	KIT	CNTLN	ADIPOQ	BRAF
PACRG	HPGD	COL1A1	SOD1	MIR141
DYNLT1	EMSY	COL26A1	KRT74	ITGA6
KIR3DL3	CRNN	COL2A1	DOP1A	IL1RL1
PLAU	PCCB	COL6A5	CD274	PRF1

DEFB1	DCD	COMMD10	IL12RB2	MIR93
DCD	IL21	COMT	PF4	GC
CD5	IPO8	COPD	GHITM	COMT
IL13RA1	PF4	COX4I2	TMPO	PPOX
TNFAIP3	IL17RA	COX5A	DAW1	CCR7
ACE	TAF1	CP	COPS7A	SLC17A5
FBN1	DEFB125	CPA3	IVL	NR1I2
GUSB	DEFB127	CPN1	PRTN3	MIR222
CD1A	DEFB132	CPO	CYP2D6	IFNA1
NCF1	DEFB124	CPS1	MAPK14	PSTPIP1
MBL2	ASAH2B	CRB1	CCL21	SLC5A2
NLRC4	LCE1B	CRCT1	LEP	TGFB2
OVOL1	DEFB128	CREB1	ADAM8	GGT1
WDR61	OSMR	CREB5	GSTM1	LEPQTL1
IL18R1	SLCO2A1	CREBBP	HDC	MIR20A
KIF3A	CCR5	CRH	CXCL12	GIP
TBC1D4	ZAP70	CRHR1	GAS2L2	TNFRSF8
PSMB8	MTHFR	CRHR2	CETP	CD28
HRAS	ORMDL3	CRISPLD2	CD1D	MIR373
ADCY10	DEFB1	CRKL	SPP1	CHD1
NLRP10	CALCA	CRLF2	SLC25A46	PRL
IL5RA	AMBP	CRP	NLRP3	PIK3CA
TNFRSF18	CXCL12	CSF1	HRH2	CD274
AQP3	PRMT7	CSF1R	BPIFA1	FOXP1
BCL11B	INS	CSF2	DNAH8	IL1R1
MAPK1	DHCR7	CSF2RB	CHIA	IFNB1
KLRB1	LRP1	CSF3	IL10RA	MIR192
KLRG1	IRF1	CSK	ORMDL3	CYP2C19
SCGB1A1	KRAS	CSMD1	IFNA1	GAD2
S100A8	IL13RA1	CSN1S1	RAG2	CPA4
ITGB2	NLRP3	CSN3	NME8	HLA-DRA
NRAS	TGM1	CSNK2B	IL12A	MMP9
MIR148B	PI3	CSTA	TLR6	VEGFA
EMSY	IL1RN	CTCF	IL21	TG
GP1BB	CASR	CTLA4	PIP	NR3C1
IGF1	ERCC3	CTNNA1	RAG1	ERBB3
HMGB1	ERCC2	CTNNA3	IL25	CYP2B6
PF4	PCCA	CTNNB1	SLAMF1	TBXA2R
TLR6	GTF2E2	CTNNBL1	C5AR1	CX3CR1
POSTN	NUP107	CTNND2	FAS	AGTR1
CASP3	SBDS	CTRL	TLR10	ARL6
IDO1	SIK3	CTSE	STK36	PF4
HLA-DMA	SRP54	CTSG	IL1RAPL2	PPARA
VEGFA	TBCK	CTSS	CHIT1	HRH4

POMC	GTF2H5	CTTN	KLK7	ADRB3
LRBA	RNF113A	CX3CL1	IL27	UGT1A6
CIITA	MPLKIP	CX3CR1	RBFOX1	DEFB4A
PNPLA1	TARS1	CXCL1	RHOA	ESR2
ZAP70	EFL1	CXCL10	CLCA1	SBDS
CRH	RNU4ATAC	CXCL12	IFNGR1	MIR150
NPY	EDN1	CXCL2	SST	PIK3CG
TBXA2R	PSORS1C1	CXCL5	HMOX1	SMAD4
MUC7	FLG2	CXCL8	ICOS	APOA2
MAP2K2	IL15	CXCL9	IL6R	SCN9A
TRAF3IP2	FASLG	CXCR1	JUN	MIR106A
NCF4	SLC35C1	CXCR2	PMP22	CD164
HLA-DPB1	AOC1	CXCR3	NTRK2	JAK2
COMT	PTPN22	CXCR4	TPMT	FGFR2
WAS	TGFBR2	CXCR5	MUC7	MMP2
IRF2	TAP2	CYBA	BRCA2	FCGR1A
CASP1	HLA-A	CYBB	RORC	CSF3
AP1S3	NAT2	CYCS	TGM3	TRPA1
BRCA2	GPHN	CYFIP2	NEK10	SLC22A5
IL23A	MAP2K1	CYP11A1	LRRC56	NRAS
DPP4	TBXA2R	CYP1A1	CFAP221	SERPINA3
ITGAL	IL17F	CYP1A2	DNAAF6	MYH11
NLRP12	ITGAL	CYP1B1	LRP1	HMOX1
CD7	ANKRD1	CYP21A2	KIT	MIR200C
LEP	CTSG	CYP24A1	VDR	CXCR4
CD207	KLK5	CYP27A1	JAK2	SV2A
MUC5AC	HFE	CYP27B1	ABCB1	BRCA1
VDR	SPINK9	CYP2C19	LTA4H	AIRE
SEMA3A	ACTL9	CYP2C9	PSIP1	HBG2
HSPD1	KATNAL1	CYP2D6	CD83	MIR15B
PTPN22	ARFRP1	CYP2E1	SCP2	MIR140
COL7A1	KNG1	CYP2J2	MUC1	MIR223
MBP	TLR6	CYP2R1	ALG12	PIK3C2A
NGFR	MBL2	CYP3A4	WNT2B	LCAT
KLK5	TOR1B	CYP3A5	CD5	EFL1
IL25	TLR1	CYP4F22	CIITA	KLK3
FAS	POSTN	CYSLTR1	IL13RA1	SOD2
TRPV3	CD1D	CYSLTR2	CCR1	PON1
CD3G	PSMB8	DAP3	AQP5	NTS
TLR3	SPRR3	DCBLD2	NGFR	TERT
FASLG	CYCS	DCLK1	CSTA	NPSR1
ELANE	CD5	DCTN4	MDH2	BGLAP
MIR126	CTRL	DDIT3	RFX5	FAS
MIR148A	LEP	DDX1	LORICRIN	MEFV

MIR152	KRT74	DDX39B	GLI3	IGFBP3
ASRT8	NPY	DDX41	ABL2	MALT1
LAMA3	FCGR2A	DDX58	EPS15	SRP54
TLR1	SMAD3	DEFB1	ENTPD6	CNR2
NR3C2	CSF3	DEFB103A	CYP2C9	MIR29A
FZD6	RAD50	DEFB103B	ENO1	KRT18
IPO8	STAT5A	DEFB4A	AHR	CAT
KRT74	PLAU	DEFB4B	TXK	TIMP1
DOCK8-AS1	CLEC16A	DEL16P13.3	HMGB1	ABCC2
KONDS	HLA-C	DEL18Q	PIK3CD	HRH2
BZX	ETS1	DEL22Q11.2	TNFAIP3	LYN
C5AR1	VDR	DENND1B	LAMA3	SULT1A1
PIK3CA	CIITA	DGKG	ADA	BLK
MMP12	GJB2	DISP1	C3AR1	NGF
NR3C1	THPO	DKK1	CXCR4	HAVCR1
MALT1	TLR10	DLL1	PPARG	HADHA
IFNA1	TLR3	DMXL2	DUOX1	MIR200B
FECH	AHR	DNAH8	MIR142	GSTT1
S100A9	IL23A	DNASE1L3	HIF1A	ACHE
CD80	MBP	DOCK1	NOD1	BBIP1
SLC27A4	GP1BB	DOCK8	NTRK1	RNASE2
SPP1	FGFBP2	DOCK8-AS1	MME	TPSAB1
ORMDL3	MSMO1	DOP1A	TMEM79	TRAPPC3
CX3CL1	CCR8	DPP10	SIGLEC5	HLA-C
SST	SEMA3A	DPP4	BCL2	PHF11
TPT1	LCE5A	DRD2	BDKRB2	CCL20
HLA-DMB	TNFSF13B	DSG1	HLA-DPA1	PTPRC
PEPD	CD80	DUPXQ28	RETN	DNAJC21
PTGS2	GNAS	DUSP1	KCNJ11	CP
CYSLTR2	VAX2	DUSP10	CTSE	IL7R
KRT5	TNFSF12	EBI3	CYP4F22	PRKCQ
STAT5B	COMT	EDIL3	DEFB103A	EGFR
IFNA2	GSDMB	EDN1	AICDA	GNLY
IRF1	BGLAP	EDNRA	RELA	AHR
CTSG	ERBB2	EDNRB	LEPQTL1	SERPINE1
PCCB	SERPINB7	EFEMP2	MAPK1	ETV5
IL12A	HLA-DMA	EGF	CRH	CLC
CSF3	IL18RAP	EGFR	MMP2	POSTN
IRF3	TNFSF18	EGR1	PBX2	NQO1
KNG1	AOC3	EHF	IL7R	EDN1
TNIP1	CASP3	EHMT1	CXCR2	ARG1
PTPRC	TNFSF4	EIF4E	CST1	UGT1A
KRT10	CYSLTR2	ELAC2	SEMA3A	CD34
HMOX1	GLB1	ELANE	ABCA1	TRMT10C

IL17RB	CBL	ELF5	FTO	CCL13
CTRL	ADCY10	ELN	PRDM16	F10
IL13RA2	HLA-DPB1	ELOVL4	DLG1	GUSB
MMP3	DDX39B	ELP1	FAM20C	NACA2
CASP8	SRC	EMSY	ST8SIA2	MMP1
C3	TNFSF13	ENO1	RBX1	MKS1
CCR8	PNMT	ENPP3	BSND	PEX14
KRT17	SLC26A2	EOE1	OSTF1	TLR1
SPRR1B	CD19	EOE2	FGF20	AR
NFKB1	SPRR1B	EP300	DYRK4	RBP4
FLI1	DPP4	EPHX1	AKR1E2	IL5RA
SLC6A4	HLA-DPA1	EPHX2	CHRDL2	UGT1A7
ITGA6	IL13RA2	EPO	FERD3L	PDE4A
CLDN1	SLC35D1	EPRS1	NKAIN3	MEN1
NOS2	SPN	EPX	SVIP	ARID1B
MIR146A	KLK6	ERBB2	SYCP2L	GH1
CXCL12	CD207	ERBB4	TUSC1	MIR34C
LDHA	CYP24A1	ERCC2	ZNF776	PMPCA
CCL28	REL	ERMP1	LINC00486	TNFSF10
CXCR4	C5	ESR1	LINC01426	P2RY12
CLEC16A	CASP1	ETS1	TPSAB1	CYP2A6
TGFB2	SST	ETV5	IL21R-AS1	MIR144
TFRC	MAP2K2	EZR	CCL8	GP1BA
SPRR1A	IL25	F10	BMP6	SDHB
TNFSF18	AKT1	F2	WDR36	ODC1
TNFSF13B	PPARG	F2R	DEFA1	H2AC18
IL18RAP	FAS	F2RL1	DPP4	CASP3
JAK3	PDGFRA	F2RL3	SEMA6A	HLA-DQA2
CXCR2	RELA	F3	SUCLG2	TBX21
CXCR1	ADAMTS10	F5	CP	SLC11A1
SOD2	CLEC7A	F8	SLPI	MAPK14
KATNAL1	KRT10	FADS2	AKT1	SCGB1A1
ACTL9	CHIA	FAIM2	GZMB	ADGRG6
FABP5	NFKBIL1	FAM13A	EPO	DPP4
PPARG	JAK2	FANK1	TMEM232	BMP6
MEFV	NGFR	FAS	IRAK4	PLA2G2A
PPARA	CXCR2	FASLG	MMP1	CHI3L1
CD209	LCE3B	FBN1	HLA-DRA	APOA1
IRF7	HLA-DMB	FBN2	CD63	MYC
TNFRSF1A	GPSM3	FBXL7	PTGDS	JUN
RETN	MIR4435-2HG	FCAR	AGT	NAT1
B2M	CLIC2	FCER1A	C3	MIR483
IL17RE	RAB39B	FCER1G	SOCS1	HADHB
FADS2	NFKB1	FCER2	TACR2	B3GAT1

TNFRSF4	SLC25A46	FCGR1A	MTHFR	SOCS3
MICU1	TMEM165	FCGR2A	TF	F9
TLR10	PTGS2	FCGR2B	IL17RB	AKT1
CD19	IGFBP3	FCGR3A	ETS1	DAO
SERPINB7	PPIA	FCRL3	BLK	TNFAIP3
ERBB2	CXCR1	FCRL6	CHGA	MIR22
LELP1	CRP	FGA	COX5A	OGA
CYP1A1	NLRP1	FGF10	ERBB3	ITGAM
PLA2G4D	DLX3	FGF2	MUC2	CASP1
PRL	PIK3CA	FGF7	TGFB3	IFNL3
DNMT1	MIR146A	FGF8	CXCL1	TYR
BGLAP	ATF6B	FGFBP2	CAT	NPHP4
MUC16	S100A10	FGFR1	SERPINA1	MIR181A1
TNFSF13	LCE3A	FGFR2	ENTPD1	MIR29C
PDCD1	PLCG1	FKBP5	JAK3	PPBP
ORAI1	RPTN	FLG	EGR1	DLAT
TIRAP	SLC6A4	FLG2	PPM1A	CLDN34
APOC1	IFNA1	FLG-AS1	DHRS7	PFN1
ITK	HMOX1	FLNA	TMEM108	ITPR3
KRT19	KRT5	FLRT2	CROCC	NPHP1
ITGA4	PRL	FLT1	RPL17P2	MADCAM1
ERAP1	C5AR1	FLT3LG	SUOX	VKORC1
DDX39B	IFNA2	FMR1	AKR1B1	ITGA2B
CYP24A1	TH2-LCR	FN1	ADAM10	ACKR1
LTA4H	TRAF3IP2	FOS	FCGR3B	ADSS1
KLK6	PBX2	FOXH1	MATN2	IL12RB1
GATA1	TSBP1	FOXO3	MIR202	CCND1
UBAC2	CRHR1	FOXP3	PLA2G2A	FYN
BCL2A1	KLK4	FPR2	FCRL3	SUOX
DSG4	KLK14	FSIP1	PVT1	MIR23A
ANXA5	ELN	FSTL1	VIPR1	MIR23B
REL	EGR2	FTL	ZAP70	TACR1
TRPV1	PFDN4	FTO	SMAD3	ADAM33
IL11	ZNF365	FURIN	OCLN	CYP3A5
CP	ADO	FUT2	C4A	AMBP
S100A2	RTEL1	FUT3	SPDEF	BTK
RAD50	MAOB	FYN	MIR21	FUT2
PIK3CD	SOD2	G6PD	LYN	TREM2
SLC29A3	TNXB	GAB1	CD19	MIR18A
KNSTRN	ZBTB10	GAL	CDH1	MIR15A
S100A10	IL11	GAPDH	ACE2	IDO1
LCE3A	ITGA4	GAS1	IRF1	CRLF2
TNFSF12	ADIPOQ	GAST	TNFSF13B	CYP2C8
TNFRSF25	GRB7	GATA2	FCAR	HLA-DQB2

FCER1G	EIF4H	GATA3	CFD	GPX3
GLB1	USB1	GBA	PRKCA	UGT1A9
TLR7	BUD23	GBE1	TJP1	PDGFRA
CYP3A4	TH	GC	AQP3	LRRC32
F2R	CD1C	GCG	LTB4R	MIR132
SHARPIN	CCL28	GCLC	HLA-DRB4	ASTN2
JAK2	KRT19	GCLM	PLAU	NOD1
IL17RD	POLG	GER	CAMK4	WAS
ENO1	IARS2	GHITM	MICA	YAP1
PDYN	NDUFAF6	GH-LCR	IL18RAP	FADS1
TOLLIP	PTPN2	GHRL	ATP12A	XK
PCCA	NLRC4	GLCCI1	MICB	IL31
CCL19	ASPRV1	GLI2	TET2	KATNAL1
HTR1A	ALG6	GLIS3	RORA	HLA-E
SMAD3	MOGS	GMPPA	MSH5	KLRD1
FADS1	SLC35A3	GNAI1	GAS5	KLRC2
ARG1	ALG14	GNB3	CHRM2	SELP
ASAH2	ALG11	GNG5P5	OSM	FCGR3B
TGM5	ALG12	GNGT1	LCK	CDSN
HLA-DPA1	IGF1	GNGT2	CD55	COX4I2
PKP2	AIRE	GNPDA2	LGALS9	SMAD3
CD160	DNMT1	GNRH1	CD3D	GNRH1
ANOS1	LTA4H	GP1BA	SCGB2A2	PDCD1
FGFBP2	TRPV3	GP1BB	SCGB2A1	CTSG
CRNN	CD151	GP6	CX3CL1	HGF
GAL	VEGFA	GP9	BCL6	IRS2
SMPD2	S100A9	GPR12	IL23A	CD86
PTGS1	CYP2C9	GPR183	TRPA1	BBS2
ARTN	MIR124-1	GPRASP1	SIGLEC8	CXCL9
AREG	CDH1	GPT	TNC	CYBA
BCL2	MIP	GPX1	PTX3	NCF4
CD2	LCE3C	GPX3	GLCCI1	SIGLEC8
JUP	NLRP10	GPX4	PVALB	TGM3
ITGB4	CCDC80	GRHL2	IL19	IL18R1
KRT14	OR10A3	GRK5	SLC26A4	CRNN
ADIPOQ	MIR1208	GRN	TNFSF10	LCK
CRHR1	LINC00824	GRP	CYP2C19	AKR1B1
ADM2	RARA	GSDMA	CCN2	BBS4
DLX3	XK	GSDMB	MUC5B	PLAU
ANXA2	CD1A	GSR	CISH	NPPA
RNASE7	MALT1	GSTA1	PHB1	HTR3A
AIRE	DPP10	GSTM1	IL13RA2	MIR142
SERPINB3	LTF	GSTM3	CLCN3	ADORA1
IL9R	CXCR4	GSTO1	PPARA	CA2

FCGR2B	IL9R	GSTO2	JAK1	TGM6
SLC39A10	CTSK	GSTP1	TAPBP	FAAH
MMP8	ATP6V1G2-DDX39B	GSTT1	TGFBR3	IVNS1ABP
IL32	PCDH1	GTF2H4	GUSB	PDE10A
SMPD1	KIAA1109	GTF2H5	EDIL3	UCP2
PNMT	UBAC2	GUSB	MIR487B	BBS5
ACKR1	S100A11	GYPA	ERBB2	BBS7
ST2	IL37	GYS1	TPO	TLR10
CASP14	ARG1	GZMA	TH	MIR19A
OPRM1	PTGS1	GZMB	TRAF6	CCKBR
ADM	TNFAIP3	GZMK	CCR2	FLNA
PBX2	ADH1B	H2AC18	APOA4	REN
GPSM3	TNFRSF6B	HARS1	KCNK2	CCL27
TSBP1	KRT14	HAVCR1	PTGER2	PON2
MIR4435-2HG	F2R	HAVCR2	PARP1	COL4A4
S100A14	GAL	HBG2	KIF3A	IL22
XK	STAT4	HCG23	NOTCH1	SH2B1
TMEM165	CD7	HCG27	HSPA4	PLA2G4A
IGFBP3	HRAS	HCRT	IFNLR1	CA5A
LCN2	TNFRSF4	HDAC1	ADM	CA5B
MMP2	ACKR1	HDAC2	IFNA2	ELAVL3
GJB2	NOS3	HDAC4	XKR6	CXCL12
SART1	PHB1	HDAC5	ADAM17	ATXN2
ALDH1A1	TTC28	HDAC7	EBI3	LTC4S
STAT5A	FABP5	HDAC9	PSMB8	ITGA4
ENO2	BHLHE40	HDC	TNFRSF14	MIR127
PSENEN	PDE4D	HERC5	CHI3L1	IL21
RPTN	EFEMP2	HFE	KDR	MKKS
NTF4	IL36G	HIF1A	KIAA1109	TTC8
MAPK3	LCN2	HIRA	IL15RA	CLEC16A
C3AR1	IL32	HLA-A	ELN	TLR3
CSF2RA	BMP6	HLA-B	ICAM2	CYSLTR1
GRP	MICB	HLA-C	ST2	ATM
GRB7	TUFT1	HLA-DOA	MYC	SDCCAG8
S100A4	RNASEH2C	HLA-DPA1	LPP	IFT27
RIPK2	HLA-DQA2	HLA-DPB1	ADAD1	SLC9A6
IL1RAPL2	ERCC6	HLA-DQA1	PSMB9	GBA
FAF1	CP	HLA-DQA2	IRF4	THPO
FUT7	OPRM1	HLA-DQB1	CARD11	PTPN3
FLT4	TRPV1	HLA-DRA	NAB2	BBS9
TNXB	ADM	HLA-DRB1	ANAPC1	PTS
VNN3P	AREG	HLA-E	UCN	UGT1A4
RELA	D2HGDH	HLA-G	RANBP6	F2RL1

ADH1B	THAP4	HLX	ZBTB10	ALK
SEC23A	MME	HMGA2	BCL2L1	GRHPR
TNFSF14	HTR1A	HMGB1	CD207	TNFSF11
MTOR	MMP3	HMGCR	CCL14	CREB1
LTF	CLC	HMOX1	IL3RA	IL6R
AOC3	MAPK3	HNMT	PAPPA	CYBB
ETS1	LRBA	HP	RBM17	BCL2L1
JAZF1	CSNK2B	HPGDS	HSPA8	CDKN2B
DEFA5	DMRTA1	HPSE2	SCG2	PIK3CD
S100A6	XIRP2	HRH1	VIPR2	TBK1
S100A1	ADAM10	HRH2	LOC101927421	ACTA2
S100A3	CD63	HRH3	DSG1	KLK7
PGLYRP3	CAT	HRH4	KCNE4	PLA2G1B
S100A5	PPARA	HRNR	NTRK3	SFTPD
EDNRB	MEN1	HSD11B2	CD44	KIF7
IL17C	MAOA	HSD3B1	PDCL	CDK1
BCR	ELOVL5	HSP90AA1	TP53	MBL2
TP53	SLC9A4	HSP90AB1	MIR146A	ANXA5
LAMB3	CDKN2B	HSPA1A	FOXO3	CAPN14
EGR2	ATP12A	HSPA1B	SERPINF1	CCL1
PFDN4	MUC1	HSPA4	DEFB1	DACT1
ZNF365	HRH2	HSPB1	HMOX2	MID1IP1
ADO	LTC4S	HSPD1	CYP1A1	CBS
CCDC80	KRT17	HTR2A	IL23R	TUBGCP3
OR10A3	EGFR	HTR3A	TBXT	NFKBIA
MIR1208	ADA	IAPP	EZH2	DPYD
LINC00824	IQGAP1	ICAM1	RETREG1	EPPIN
UBASH3A	SP1	ICAM3	FTH1	IVL
ATF6B	PTPN11	ICOS	SLC35D1	CD44
BLMH	NDUFAF1	ICOSLG	PDGFRA	EPHX2
MIR223	NELFA	IDO1	MYDGF	CDH1
CDH1	CD53	IDS	ALOX15	PTPN1
MMP10	TSPAN32	IFIH1	NFKBIA	SCN1A-AS1
CHIA	NLRP12	IFNA1	TET1	IL17F
NAMPT	CYP27A1	IFNA2	HSD11B1	CFTR-AS1
MYD88	KRT75	IFNAR1	HSD11B2	SLC22A4
DEFA4	ARNT	IFNB1	BCL2L12	PRODH
DEFA6	TNFRSF10A	IFNG	IL17RA	NRL
TIMD4	PHLDB1	IFNGR1	NOX4	NCF4-AS1
SPRR3	JUN	IFNGR2	NOX1	CHIT1
ADAMTS10	HLA-DRA	IFNL1	DEFB104A	TRB
ZBTB10	PNPLA1	IFRD1	GSDMB	F5
ATP6V1G2-DDX39B	PLAUR	IFT172	PRMT1	ENO3

HSPB2	WIF1	IFT27	CYP2R1	PCSK7
S100A12	COL10A1	IFT74	MPZ	UQCRB
KYNU	CRY2	IGES	TRPC1	FUT1
MRGPRX2	COL8A1	IGF1	CDX1	ZFYVE16
EPO	CLEC4E	IGF2	RNF39	B3GALT1
ALG6	IL17RB	IGFBP3	TRPV4	OSER1
MOGS	NTF4	IGHE	GJB2	OCM
SLC35A3	IKBKB	IGHG1	LCN1	CACTIN
ALG14	RUNX1	IGHG3	MRPL4	SAGE1
SLC35D1	RORC	IGKV2D-29	MC1R	GSTA1
SLC35C1	ST2	IGSF3	MIR143	TAP2
ALG11	CHIT1	IKBKB	AGER	TGIF1
ALG12	MAP3K11	IKZF2	ID2	TLR5
APOA1	CASC3	IKZF3	RMI2	TMEM79
CLDN4	GSDMA	IKZF4	HLA-DQA2	MMP3
CYP27A1	WIPF2	IL10	CHRM3	PNPLA3
TYK2	LINC00299	IL10RA	CLDN7	CASP9
TMEM232	GVQW3	IL10RB	BDKRB1	SLC5A4
BMP6	LINC02757	IL11	ATF6B	SP1
JUN	LINC02676	IL12A	CST3	ADORA3
KRT9	ENSG00000255135	IL12B	CSF2RB	ADORA2A
ADA	ENSG00000223808	IL12RB1	RTEL1	ITGB3
COL6A6	LOC107984360	IL12RB2	PHLDB1	TPSD1
NELL2	ENSG00000254810	IL13	CTSG	NCF2
TRPC6	ENSG00000254755	IL13RA1	PITX2	DNAH5
GNAI1	MK280269-012	IL13RA2	PPP1R10	DNAH9
TXN	piR-50308-096	IL15	MIR15A	CCDC40
CD163	CYP21A2	IL15RA	FBN1	DNAAF1
AOC1	CLIC1	IL16	MEFV	CFAP298
IRF5	S100A14	IL17A	A2M	DNAAF11
ERAP2	TYK2	IL17D	SLC18A3	ODAD4
NAT1	TLR5	IL17F	IL1RAPL1	CYBC1
MIR203A	TPD52	IL17RA	NFATC2	CFAP300
BHLHE40	CASP14	IL17RB	NTF4	AFP
SLC25A46	IL17C	IL18	INS	HIF1A
IKBKB	ALOX5AP	IL18BP	DOCK8	IL12RB2
PDE3B	CPA3	IL18R1	ERBB4	FOXP2
NTS	PIP	IL18RAP	IL36G	CDKN3
IL2RG	ITGA6	IL19	PON1	DEFB103B
HTR2A	ADM2	IL1A	TIRAP	ALOX5AP

CD36	TGFA	IL1B	FCRL5	GLDC
CRP	LAMA3	IL1R1	FAM167A	GABRE
TRAF6	TMEM232	IL1R2	MIR495	LPO
MME	SKIV2L	IL1RL1	C5	GALC
IL24	G6PD	IL1RL2	NOTCH2	AFF4
ABCG2	GHRL	IL1RN	MIR149	BANCR
SLC26A2	PVALB	IL2	LOC102723407	IL13RA2
CRISP3	NAGLU	IL20	HLA-DRB5	PHYH
CAT	CRTAP	IL21	HLA-DQB2	PRTN3
SH3KBP1	ANXA5	IL21R	CYP21A1P	AGT
LTC4S	CLDN4	IL21R-AS1	HLA-DRB6	ABCC1
PECAM1	B3GAT1	IL22	HLA-DQB1-AS1	DSPP
ERBB4	ACP1	IL23A	BAK1	HTR1A
MMP13	RAB5B	IL23R	DAXX	C3
OXA1L	ADAMTSL4	IL24	ARG1	PVT1
IL17RA	NBR1	IL25	TNFRSF18	PIK3R1
FKBP1A	CSF2RB	IL26	DPP10	MOGS
UROS	ANAPC1	IL27	WNT5A	RUNX2
MUC1	PSMC3IP	IL2RA	CALCRL	LCN2
MC1R	CAVIN1	IL2RB	CD244	MAPK3
MC3R	IKZF3	IL3	SERPINB1	PRNT
DPEP1	JAK3	IL31	MIR31	HSPA4
PPARD	RORA	IL31RA	MIR181A1	PROP1
CCR1	SYNGAP1	IL33	SLC22A5	PTGER4
DNAJB6	SETDB2	IL37	ATP4A	ADAD1
NCR3	TNFRSF25	IL4	GATA3-AS1	TNFSF13B
CLDN3	MMP12	IL4R	F3	PI3
IL19	KYNU	IL5	HTR2A	PCNA
HSD11B1	HCG27	IL5RA	ATF3	MT-ND2
SPINK9	TXN	IL6	CD68	PARP1
LAMC2	NFATC2	IL6R	TWIST1	CDH26
PIP	TERT	IL6ST	HDAC11	EPC2
IQGAP1	SPRR2B	IL7	DMBT1	ADM
FLII	AR	IL7R	ASIC3	DEFB1
GJA1	ALOX15	IL9	UBE2Q1	MIR199A1
MTHFR	RIPK2	IL9R	SFRP5	MBP
ABCB1	TP53	INHBA	NEAT1	U2AF1
NR4A2	TFRC	INPP4A	MIR223	ETS1
F2RL2	SERPINB3	INS	MIR338	COX5A
F3	RXRB	INSIG2	FOXD3-AS1	MIR28
TLR8	BRD2	INSR	RPL21P119	MIR574
RORC	WNT11	IPO13	RPS14P8	FLG-AS1
ERCC6	C4A	IPO8	TIMD4	PTGDR
ATP12A	AGER	IRAK1	PTHLH	HLA-DRB5

TRPA1	PIP5K1A	IRAK3	IKZF3	NDUFS2
DMRTA1	AGPAT1	IRAK4	OXA1L	NDUFAF1
XIRP2	SETDB1	IRF1	SLC22A2	CHIA
NPM1	IL15RA	IRF1-AS1	POU2AF1	MYD88
SLC2A2	MSH5	IRF2	TNFRSF10A	ALMS1
AVP	SYVN1	IRF3	PTAFR	TNFRSF18
SGPL1	SIPA1	IRF4	MUC4	HRH3
CLEC7A	CATSPER1	IRF7	IFNL1	MIR9-1
NR4A1	HLA-DRB5	ITGA2	PLAT	IL32
CYP2C9	HLA-DOA	ITGA4	PLA2G6	RBFOX3
GNA11	HLA-DOB	ITGAL	VAV3	GRIN2B
PRF1	THEM4	ITGAM	TNS1	CAMP
COIL	SNX27	ITGAV	SIM2	MIR196A1
SSTR5	CCHCR1	ITGAX	GSDMA	ANPEP
CD27	CTSW	ITGB1	TAFA2	SLC27A1
VPS50	HLA-DQB2	ITGB2	LY6G5B	GLB1
HBEGF	MRPL11	ITGB3	PTGER4	DBH
PHB1	PPT2	ITGB4	SHH	SNCA
OPRK1	PHOSPHO1	ITGB7	BRD2	ERBB4
IFNK	SNX32	ITK	HLA-DMA	IFIH1
CABIN1	RBM14	ITLN1	HLA-DMB	SLPI
SP1	ZNF687	ITPR3	HLA-DOA	GAPDH
DSC3	ZNF652	IVL	HLA-DOB	TMEM165
CHIT1	ZFPL1	JAK1	VPS52	ITGA2
S100A11	VPS52	JAK2	GVQW3	RPE65
THY1	EGFL8	JMJD1C	RPS3AP21	SERPINB2
MIR151A	MRPL9	JUN	LINC02757	CTRL
CTSL	TESPA1	KANK1	ENSG00000255135	MIR125A
LGALS9	GPANK1	KCNJ11	BCLAF1P1	RUNX1
ACP1	EHBP1L1	KCNMB1	LOC107984360	CTNNA3
TH	RMI2	KCNN4	ENSG00000254810	CCL18
MBTPS2	AP5B1	KCNQ1	ENSG00000254755	S100A7
GSDMB	GPATCH1	KCTD15	MK280269-012	ACP5
AR	LY6G5B	KDM4C	RF00017-4442	BCL2
KLKB1	TMPPE	KDR	HSALNG0044120	FMR1
YAP1	TSGA10IP	KIAA1109	HSALNG0044122	TMPO
DSG2	MUC22	KIF3A	HSALNG0049431	MIR30A
TNFSF10	HCG22	KIF7	piR-50308-096	NFKB1
AKR1C3	CYP21A1P	KIFC1	NR1H2	TSPO
MAPK14	GATA3-AS1	KIT	GLB1	NFE2L2
MDH2	HLA-DRB6	KITLG	MSX1	TH
SLC7A9	HCG23	KLF2	TUFT1	PLOD1
SPI1	FLJ40194	KLHL5	ITSN2	MIR10A
PLS1	HLA-DQB1-AS1	KLK11	CSNK2B	UGT2B15

DDX39A	OVOL1-AS1	KLK3	SKIV2L	ITGB2
FUT4	TH2LCRR	KLK5	ANXA5	SLC22A2
KPRP	KRT8P26	KLK7	STAT5A	TAP1
RPS5P5	STK19B	KNG1	CCR9	TBX5
PPL	SOCAR	KPNB1	CD164	APOH
CA3	HNRNPCP4	KRT1	DGKE	ALG14
VIM	LINC02571	KRT13	SPTBN2	AGER
DES	SLC25A38P1	KRT14	SLAMF8	ALG6
PLA2G4A	RNU6-1213P	KRT18	EPRS1	MUC5AC
LTB4R	LOC101928272	KRT19	ADCY10	NTF3
HRH2	ZNF652-AS1	KRT8P16	EP300	ALG12
BCL10	ENSG000002242<br>28	KRTCAP3	CREB1	TGFA
KIAA1109	ENSG000002343<br>89	LALBA	UBAC2	FOXO1
SERPINA1	ENSG000002620<br>39	LAMP1	RTF1	PTGES3
TNFRSF10A	ENSG000002509<br>48	LAT	DDX39B	MIR31
CD5L	ENSG000002548<br>55	LBP	SLC6A4	MVK
TSPO	ENSG000002487<br>14	LBR	LEPR	CD80
MIR483	ENSG000002550<br>38	LCE3B	ANXA1	MMP12
CD44	AIMP1P2	LCK	ARSB	KRT7
KLRK1	MTND5P15	LCN2	MRC1	PTEN
FGFR2	lnc-TNFSF18-1	LELP1	PMP2	CYSLTR2
VEGFC	lnc-GATA3-19-0<br>01	LEP	IL6ST	SERPINF2
ASPRV1	lnc-GATA3-19-0<br>02	LEPQTL1	MAPK8	PGR
SHOC2	lnc-GATA3-20	LEPR	CD2	STAT5B
CNFN	lnc-HLA-DRB1-<br>8	LGALS1	TGIF1	SH2B3
B3GAT1	lnc-KIN-10	LGALS3	FADS1	BLVRB
PLAUR	HSALNG004942<br>7	LGALS9	FADS2	COG7
GSN	HSALNG004942<br>8	LIFR	SERPINB3	MIR149
RAB7A	HSALNG011717<br>6	LIG4	MAP2K5	S100A8
CHGA	HSALNG004942<br>9	LMX1B	POU5F1	MIR34B

LGMN	piR-44329	LOC100287329	NOTCH4	JAK3
MCC	piR-36419	LOC101928940	PFKFB3	IL17D
CST6	RF00017-5040	LOC106050102	DHX16	SOST
CTSV	lnc-FLG2-1	LOC107303343	MDC1	CD46
LGALS7	LOC107986073	LOC110806262	ABCF1	PSIP1
CKLF	PKP1	LOC110973015	AAGAB	LRP1
JCHAIN	NOTCH4	LOC111365141	LSM2	CBLIF
STIN2-VNTR	DSG4	LOC111674463	XK	SORD
LOC110806262	C3AR1	LOC111674472	TIPIN	PDGFA
ELP1	TNC	LOC111674475	CCHCR1	MIR29B1
TNFRSF14	TONSL	LOC111674477	NFKBIL1	CNOT6
RELB	EPO	LOC113633877	TCF19	MIR214
AZU1	CYP1A1	LOC113664106	NRM	SOCS1
ITSN2	NEUROD2	LORICRIN	SKOR1	IL12B
CA2	CDK12	LOX	ZWILCH	NR1I3
TNC	MED1	LPA	IQCH	LPP
ACKR2	MED24	LPO	PSORS1C2	DAB2
LINC02676	PLA2G4A	LRBA	C6orf47	RANGAP1
ENSG00000223808	MIR223	LRG1	HCP5	F8
OSM	SERPINA1	LRP1	SFTA2	MIR139
KLK4	RAC2	LRRC32	MUC22	MIR486-1
KLK14	LDLR	LTA	PSORS1C3	MIR423
IL23R	FUT2	LTA4H	HCG27	CLCA1
PTK2	SDHC	LTB	HCG22	ICAM3
MC5R	NDUFS2	LTB4R	SNORD16	SULT1E1
CLDN7	APH1A	LTB4R2	MIR6891	ADCYAP1
HAS1	BACH2	LTBP1	STK19B	MAPK1
APOE	RPS25	LTBR	RPL3P2	VWF
SPINT2	ADAD1	LTC4S	ENSG00000272501	TLR7
HAS2	RSL24D1	LTF	HNRNPCP4	GLA
VAMP3	PUS10	LY86	RNU6-1213P	ADRB1
CD180	NCAM1	LY96	ENSG00000212228	KMT2A
KRT75	GALR1	LYN	ENSG00000263033	MIR124-1
HP	SERPINB8	LZTFL1	ENSG00000260773	ADORA2B
PKP1	GJC2	MAF	ENSG00000272221	ABCB11
NOS3	TSHZ1	MALAT1	ENSG00000272540	LSM4
DSC2	SALL3	MAP3K7	lnc-HLA-C-2	MIR24-1
GAST	ZNF516	MAPK1	ENSG00000271581	MIR193A
NCAM1	ZNF236	MAPK10	GTF3AP1	TMEM37
IL10RB	TULP4	MAPK14	NONHSAG043472.2	NBN
UCHL1	LTB4R	MAPK3	lnc-HLA-DQA1-8	CLEC7A
NTRK1	TNIP1	MAPK8	lnc-HLA-DRB1-6	ITGAE
STIM1	STIM1	MAVS	lnc-IQCH-5	HAVCR2

TP73	TGM2	MB	HSALNG0049258	EFEMP2
ENPP2	PDGFRB	MBL2	HSALNG0106744	PLG
UGT1A9	MIR203A	MBP	NONHSAG043568.2	LBP
SATB1	HTR2A	MC4R	MN298114-181	ITGAX
TIMP2	PVT1	MDC1	NONHSAG017238.2	GJA1
AZGP1	TPSAB1	MDM2	piR-47864	LORICRIN
FKBP8	PGAP3	MED24	MK280269-056	KDM4C
SRR	PDCD1	MEFV	piR-30396	IL11
SDC4	POGZ	MEG3	piR-47234	CRYAA
VDAC2	CLIC4	MEN1	piR-52740	CYP1B1
ESD	ITLN1	MGAM	CYP3A5	HSD11B1
HAS3	FGF7	MIA3	ADRA1A	ARG2
PITPNB	GAST	MICA	AHI1	ERCC6
CLIC4	NOS1	MICB	IFT172	SERPINC1
CRISP2	INPP5D	MIF	CEP89	ALG11
ESM1	MMP2	MIR122	H2AX	SLC35A3
ORM1	NAB2	MIR124-1	IFT46	SLC35C1
TTF2	GP9	MIR125A	PFN1	HMGB1
TIMP4	GLA	MIR125B1	XCL1	LMBRD1
ITLN1	MYB	MIR126	MBL3P	NTRK2
HS3ST2	ESR1	MIR140	LGALS1	MIR200A
APLN	MTOR	MIR142	IL24	MIR210
NAT9	TJP1	MIR145	GPT	MIR125B1
TIGIT	CXCL5	MIR146A	FCGR2B	EMCN
RABGAP1	CCL19	MIR148A	PAK2	LGALS9
ADGRE1	F2RL3	MIR148B	HFE	IL31RA
CALML5	BCL6	MIR149	FGL2	CTSE
ACKR4	APOE	MIR152	MYOM2	DEFB103A
ADGRE3	GSN	MIR155	FAM76B	SULT1A3
NAP1L2	MYLK	MIR15A	FLG-AS1	TNFRSF1A
LILRA6	POU5F1	MIR15B	MMP8	KLK5
VSTM1	EHMT2	MIR16-1	PNMT	TRAF4
SBSN	PI4KB	MIR192	IGF1R	VTN
OR10G7	TBL1XR1	MIR196A2	EGF	TNFRSF11A
KIR2DS1	C4B	MIR199A1	TNFRSF1A	LINC02757
MIR194-1	TCAP	MIR19A	COL21A1	EPRS1
PPBPP1	DHX16	MIR19B1	FAM114A1	CRHR1
RAG1	EFHC1	MIR200A	MIR574	IL37
SLC17A5	MDC1	MIR200B	RAD50	MMADHC
CD247	SLC6A15	MIR200C	MTNR1A	GPA33
HSPA1A	SLC9A2	MIR203A	IFNB1	SYT8
TJP1	ABCF1	MIR21	GPR183	CD1D

ARNT	LSM2	MIR22	KCNH2	MIR92A1
DHCR7	UBE2Z	MIR221	STAT5B	DSC1
RIT1	STK19	MIR222	CD160	DGKE
RAG2	ATP6V1G2	MIR223	ADORA3	TGM1
SLC9A4	FBXL20	MIR23A	INPP5D	KLK6
PSAP	MICA	MIR26A1	CDHR3	ALG9
FUT2	PPP1R10	MIR26A2	S100A12	NTRK3
BACH2	THEMIS	MIR28	NFIA	ABCC3
ADAD1	GABPB2	MIR29C	TNFSF18	PRKCZ
ADH1C	PAQR8	MIR30A	LINC00299	MME
FGA	TCF19	MIR31	TGM1	SERPINA6
F2RL3	TMTC2	MIR338	CDKN2A	RAF1
CDKN1A	SERPINB10	MIR499A	P2RY12	AHSG
THEMIS	BAG6	MIR98	TIMP2	CFI
lnc-FLG2-1	TRAM2	MIRLET7A1	PPIG	ATG16L1
SLC39A14	DXO	MIRLET7B	APOA1	TXK
SLC39A8	MIEN1	MKI67	LTB4R2	CYP4F22
SLC39A13	NRM	MKKS	CD1A	NUP133
GJC2	PRUNE1	MKLN1	SLCO1B1	MIR182
IL36G	C1orf56	MMADHC-DT	CTRL	MIR455
HSPG2	PSORS1C2	MME	HRH3	MIR338
CCR2	VWA7	MMP1	MIR302E	CD9
ITCH	C6orf47	MMP12	MTOR	ADCY10
CLC	HCP5	MMP19	LACTB	OFD1
CXCL1	MCCD1	MMP2	LSM4	CXCR2
MAF	SFTA2	MMP21	AR	AREG
NELFA	PSORS1C3	MMP28	CD34	MCM6
ALOX5AP	SNORD117	MMP3	RTP3	IL10RB
AIF1	SNORA21	MMP7	MS4A1	TPI1
PLEC	SNORD124	MMP8	CD93	CSF1
CYP2C19	LINC00709	MMP9	PLA2G5	TNFRSF10A
TPSAB1	MIR6891	MMRN1	CXCR5	LRBA
LPIN2	MICB-DT	MPLKIP	RYBP	MIR151A
CD151	TSBP1-AS1	MPO	TNFSF8	MIR335
CYP21A2	PPIAP9	MPP7	IL17C	SPP1
MICB	RPL3P2	MPZ	LRG1	S1PR1
HLA-DQA2	ENSG00000272501	MRC1	TERT	SLC26A2
OCLN	MIR5708	MRGPRX2	SETDB2	KLK4
RARRES2	UBE2D3P3	MS4A1	GNLY	SPTBN2
NACA2	RPL15P4	MS4A2	PRKCQ	DCD
NLRP2	ENSG00000250751	MTCH2	RXRB	KLK14
CARD8	ENSG000002664	MT-CO2	AGPAT1	SLAMF8

	69			
NELFCD	ENSG00000254975	MTHFR	MANBA	SPINK9
MIR144	ENSG00000262837	MTNR1A	BDH2	PTGES
TGFA	ENSG00000272221	MTOR	OVOL1	HRAS
RNASEH2C	ENSG00000272540	MTOR-AS1	GPSM3	HLA-DPA1
ALDH2	RN7SL335P	MUC1	ICE2	SERPINB4
ATP2A2	lnc-HLA-C-2	MUC15	AP5B1	POR
NOS1	ENSG00000271581	MUC2	LINC00298	ENTPD1
CASR	lnc-MIEN1-1	MUC22	CASC11	CXCL11
ITGB3	NONHSAG043472.2	MUC4	HCG23	RUNX3
CLIC1	lnc-HLA-DQA1-9	MUC5AC	MIR1204	SLC12A3
RORA	lnc-GRB7-2	MUC5B	ID2-AS1	PLCG1
ESR1	lnc-MFSD9-8	MUC6	TH2LCRR	GLI2
PTPN2	lnc-HLA-DRB1-7	MUC7	SRRM1P1	SLC6A2
NPSR1-AS1	HSALNG0049245-002	MX1	RNVU1-32	URB2
PPIA	HSALNG0049423	MYB	SLC25A38P1	ARNT
AGT	HSALNG0049424	MYC	ENSG00000224228	PNMT
IFNB1	HSALNG0049258	MYCN	ENSG00000260651	PDGFRB
TG	HSALNG0085862	MYDGF	M31519	FN1
SELPLG	MG828730-053	MYH11	lnc-ICE2-7	ARSB
ADAM10	piR-48841	MYLK	lnc-HLA-DRB1-8	RECK
ANXA1	MN298114-181	NACA	lnc-NEMP1-2	COL4A1
CCND1	piR-47864	NAT1	HSALNG0049427	MPL
NBAS	MK280269-056	NAT2	HSALNG0068476	EXOC4
PCDH9	piR-52740	NCKAP1L	HSALNG0049428	SKAP1
SCAPER	RF00017-5032	NCOA1	lnc-FAM84B-8	DLX2
LGMNP1	LOC105369875	NCOA2	HSALNG0106356	ANGPT4
ITGA2	HSALNG0092788	NDFIP1	HSALNG0012816	CHCHD3
PDGFRA	HSALNG0085863	NEAT1	HSALNG0049429	SERPINB10

BCL6	GATA1	NEDD4L	piR-51710	RHOBTB1
NFATC1	SDHB	NEGR1	RF00017-4021	RNASE11
NFATC2	HP	NEK9	RF00017-5040	RNASE9
MIR124-1	ATP4A	NEU1	CR2	LINC00845
NFKBIA	HBG2	NFATC1	TREM1	ST13P7
IKZF3	SPINK6	NFATC2	CD200R1	RNU6-92P
PRKCA	NTS	NFE2L2	SERPINA6	HSALNG0122068-001
ASGR1	VNN3P	NFKB1	PLA2G4A	piR-32023-098
THPO	LTBP1	NFKB2	LPO	AQP2
TOR1A	SEC23A	NFKBIA	TEK	COL3A1
GHRL	MYC	NFKBIL1	REL	GP1BB
ATP4A	GRM4	NGF	VDAC1	IL9R
EDA	BCL2L11	NGFR	POU2F1	SLC2A5
TRPV4	MPST	NIPAL4	GBA	RYR1
MICA	PRPF6	NKX2-1	MIR17	ITK
AGER	ATP8B2	NLRP3	P2RX7	IL18RAP
PROS1	ICAM5	NNMT	CLDN1	C16orf72
ITGA1	PPP2R3C	NOD1	SH2B3	MAPK8
KIR2DL3	DIDO1	NOD2	PDCD1LG2	MAP3K14
HLA-DRA	FAM177A1	NODAL	BLVRB	CXCL1
CTSS	SLC2A4RG	NOS1	UNC119	CTSD
LIFR	UCKL1	NOS2	SFTPB	ATG5
PTAFR	UBAP2L	NOS3	FCN2	MIRLET7D
GP9	TUBG2	NOTCH4	IKBKG	FOXJ1
CNTF	ZBTB46	NOX3	ABCC1	MTRFR
PTGER2	LIME1	NOX4	ALDH2	CEACAM5
EDNRA	KRTCAP2	NPHP1	SMAD4	SMPD1
CREB1	TNP2	NPPA	RARA	CD209
ACY1	PRR5L	NPPB	TRAF3	TFF1
CXCL2	ZNF704	NPS	BCL2L11	BCL10
PRMT7	SUCO	NPSR1	TPD52	TRPM8
SERPINE1	CD200R1L	NPSR1-AS1	SESN3	IFNA2
ANKRD1	ZGLP1	NPY	PLCL1	TRA
CTCF	PRORP	NQO1	MLN	RAPGEF3
PUS10	TEX33	NR1H2	CD200R1L	ADRA1A
FGF7	PRM3	NR3C1	TTC6	COL5A1
TNFSF8	RETREG3	NR3C2	SAPCD1	ADA2
A2ML1	SAPCD1	NRG1	ECM1	HERC2
SERPINB2	NBR2	NRIP2	DUSP1	DDX58
BTNL2	RTEL1-TNFRSF6B	NSD1	NFATC1	BCL2L12
GALR1	MIR647	NSUN2	CD163	MPZ
SERPINB8	SENCR	NTF3	SPI1	MIR342

TSHZ1	ETS1-AS1	NTF4	CNR2	SLC9A2
SALL3	LINC02098	NTRK2	GGT1	F2R
ZNF516	HSD17B1-AS1	NTRK3	MAPK3	FGFR1
ZNF236	ALDH7A1P4	NTS	CFL1	CCDC26
TTC28	ENSG00000212228	ODAD3	ESR1	MRPL58
TULP4	ENSG00000225744	ODC1	PRKCD	TAS2R39
CPA3	ENSG00000263033	OIP5-AS1	NAGLU	STAT5A
VNN2	ENSG00000255060	OPN3	CRHR1	ACOT11
SEMA4F	ENSG00000267758	OPRM1	KLK6	CXCR1
HCG27	ENSG00000254027	ORAI1	PRL	IL26
CYP11B1	ENSG00000229299	ORMDL2	TYRO3	GSS
KRT6A	ENSG00000229971	ORMDL3	FIP1L1	CPA5
AIM2	ENSG00000253238	OSM	SERPINC1	LOX
ANAPC1	ENSG00000262020	OXA1L	NPPA	IL18BP
CMKLR1	ENSG00000285016	P2RX7	PTGER3	PNP
EMCN	ENSG00000280878	P2RY12	PDC	CCN2
KRT7	ENSG00000237371	P2RY2	KIF17	C4A
HBG2	ENSG00000267340	PAFAH1B1	PRF1	IL12A
ITGB1	lnc-FLI1-3	PARP1	MAG	LIF
MIR21	ENSG00000285551	PBX2	ITGB1	S100A4
IFN1@	lnc-ETS1-8	PCDH1	ST6GAL1	MUC4
DPP10	HSALNG0135257	PCDH12	GM2A	RMRP
CD63	ENSG00000285837	PCDH15	SQSTM1	KLF4
MIP	LOC105373023	PCDH20	TNFRSF10D	KCNH2
ABCC6	ENSG00000267681	PCNA	ADAM12	ADH1B
FGFR1	HSALNG010957	PDCD1	MYB	COL1A1

	6			
PDGFRB	HSALNG0088091-002	PDE10A	MERTK	SLC24A5
NF1	HSALNG0088094	PDE3B	CD247	MIR424
KCNJ1	HSALNG0028060	PDE4A	PSEN2	INPP5D
GAPDH	HSALNG0078328	PDE4B	FLI1	FBLN5
SRC	MN296981	PDE4D	FOXO1	MIRLET7B
RUNX1	piR-45012-054	PDE5A	NCF4	PITX2
MAP3K7	piR-50437-440	PDE9A	MAP3K11	SIGLEC5
KLK3	RF00017-6730	PDGFB	RUNX1	H2AX
SDHB	LOC105378327	PDGFRA	PTPN12	WT1
TIMELESS	LOC105371081	PDP1	ATF6	SPEF2
SFTPD	LOC107984408	PECAM1	CSK	CBL
IL1F10	HSALNG0066497	PEPD	CEBPA	GLO1
IL1RL2	piR-43106-052	PEX5	PAX2	SLC35D1
SMS	piR-38872-001	PF4	PPP2R1B	OCA2
CXCR5	RF00017-6733	PGAP3	PCYT1A	ANKRD26
GNRH1	RF00017-6735	PGM3	AQP2	UGT2B4
ALAS2	RF00026-1060	PHB1	AKR1C2	S100B
CPOX	SOS1	PHF11	ACADS	SMAD2
PRKCB	SOS2	PHLDB1	DDX6	CYP7B1
CTSD	LZTR1	PI3	HSD17B4	CUL4A
CASP7	A2ML1	PIK3C2A	NCSTN	ABCB10
MAOA	ERAS	PIK3CD	PPP1CB	PROZ
ELOVL5	FLII	PIK3CG	PSMA6	RAB4A
CD53	UBASH3A	PIN1	SIN3A	SLC39A10
TSPAN32	POU2F1	PITX2	SKI	IQCE
SKIV2L	TNFRSF11A	PKDCC	CLDN14	SSBP3
MDC1	NPSR1-AS1	PLA2G10	KLF5	DDIT4L
SLC6A15	TGFB3	PLA2G2A	KCNE3	RHOU
PSORS1C2	MAPK1	PLA2G4A	KMT2A	SNRPB2
HLA-DRB5	MRGPRX2	PLA2G6	PSMB7	DCHS2
DUSP1	ERBB4	PLA2G7	TNFRSF11A	EML6
CXCL16	SLC22A5	PLA2R1	SMARCE1	H3-4
IFNL1	SLC22A4	PLAG1	SDHC	CCSAP
ERV9-1	ANXA6	PLAT	TCF7	TMEM243
PTGDS	WDR36	PLAU	APOA2	TP53TG1
SOS1	MAF	PLAUR	APOA5	TMEM78
F2	DSP	PLCB1	KLF6	SSBP3-AS1
TF	FGFR2	PLCG2	ITPKB	LINC02436

CACNA1B	CYP3A4	PLOD1	EGR2	HMGN2P19
ALOX15	GNRH1	PLXNA4	DPM1	LINC02784
PIK3CG	TNFRSF1A	PMEL	CELF2	LINC02814
CXCL5	KRT7	PNMT	IQGAP1	LINC02815
PCDH1	TNFRSF14	POLI	PCSK7	ENSG00000269125
WIF1	SFTPD	POMC	P2RX4	EXTL2P1
COL10A1	MAPK14	PON1	LITAF	LINC02727
CRY2	B3GALT6	PON2	PYGB	RNU6-67P
COL8A1	MMEL1	POSTN	PROK2	ENSG00000233920
CLEC4E	IL17RE	POU2F1	NDUFS2	GYG1P2
POLE	ANXA1	PPARG	SLC22A4	RPL7AP13
ERCC3	CA3	PPBP	RUNX3	DNAJC17P1
ERCC2	CAPN14	PPIA	NRIP1	ENSG00000286150
TAF1	CD2	PPL	PIP5K1A	LOC101927947
GTF2E2	CD44	PPP1R12A	SIK2	HSALNG0078267
NUP107	IFNB1	PRG2	SYNGAP1	HSALNG0085865
SBDS	MIR31	PRKAA2	SPRY4	HSALNG0085864
SIK3	CST6	PRKCA	PTPRK	lnc-EMCN-3
SRP54	CD247	PRKCE	ADNP	HSALNG0003760
TBCK	CEBPA	PRKCQ	ADAMTS4	piR-61945-023
GTF2H5	RAG1	PRKCZ	DDAH2	LOC102723935
RNF113A	TNFSF8	PRKG1	RNF168	LOC105369539
MPLKIP	PRF1	PRL	RGS14	LOC105377345
TARS1	CD36	PRNP	ZFP36L1	HSALNG0036046
DNAJC21	CTSL	PROC	CLK3	PSMD3
EFL1	CABIN1	PROS1	ABHD12	HAO1
RNU4ATAC	CNTF	PRRC2A	AFF4	FGF2
CDHR3	PTGER2	PRTN3	ABCB5	GRIA1
IL1RAP	CDHR3	PSAP	ALG2	TMPRSS6
MIR31	MB	PSIP1	ALG9	SLC26A4
CST3	THY1	PSMB8	ANKH	EIF4E
GP1BA	CLDN7	PSMB9	BDH1	ADH1C
PLS3	HAS3	PSMD3	ATXN2	CFL1
SLC22A4	NELL2	PSORS1C1	BACH2	CERT1
MMEL1	FGA	PTAFR	CEP57	IKBKB
PLA2G2A	ARG2	PTCH1	ITPKA	GATA1
FKBP5	COL5A1	PTEN	MPST	CDK2
LAT	AGXT	PTGDR	MFAP2	CEP290
EGR1	TET2	PTGDR2	PTTG1	DENND1B
MYLK	KIF1A	PTGDS	PRPF6	LCN1
ARG2	MTRR	PTGER1	XPNPEP3	BACH2
CTNNA2	COL5A2	PTGER2	RING1	KIAA1109
CALB2	PLS3	PTGER3	SULT1A1	OPN4
REN	PLOD1	PTGER4	SUFU	RYR2

APTX	FKBP14	PTGES	SULT1A2	TNFRSF25
LZTR1	STS	PTGES3	TNFRSF6B	CCL8
MAP3K11	SQSTM1	PTGFR	RPS26	HDAC4
CHD7	NLRP2	PTGIR	SETDB1	NTRK1
KRT8	SIGLEC8	PTGS1	RAB5B	NOTCH1
FERMT3	DENND1B	PTGS2	RERE	ZEB1
DENND1B	KLK3	PTHLH	ADD2	CLDN2
CISH	MEFV	PTK2	D2HGDH	STAT4
LIF	COQ5	PTPN22	CD6	IKZF1
SQSTM1	BMP4	PTPRC	KIFC1	ITPA
SEMA4D	LGMN	PTPRD	ITGB8	TNFSF8
TNFSF15	CTSV	PTPRE	FOSL2	CACNA1H
PLCD1	GAPDH	PTPRT	GFRA3	CHRM3
HACE1	ITGA2	PTX3	PHF1	S100A9
LIN28B	CXCL1	PVT1	NDUFAF1	TFF3
RAC1	MAPK10	PYHIN1	VTI1B	COG6
RAF1	MGMT	RAB11FIP2	RPS6KC1	PRKCD
PTPN11	PRKCQ	RAB39B	RCOR1	TAC3
CARMA1	SMARCA4	RAC2	RGS19	IL1RAPL2
BIMP3	ITPR1	RAD50	VPS11	RPLP2
PPBL	MFN2	RAF1	TREH	ATP7A
BENTA	PRMT1	RANBP6	CBLN1	CALCOCO2
IMD11A	ATF6	RAP1GAP2	ARHGAP15	TSHZ1
	CAMK4	RAPGEF3	CLIC4	NOTCH4
	ACADS	RASAL2	ATG4B	CHRM2
	LIG1	RASGRP4	B3GALT4	IFT74
	DDX6	RBM17	ABCB9	PECAM1
	KAT2A	RCBTB1	ACTR1A	TNFRSF6B
	NCSTN	RCCD1-AS1	BTN3A1	MGP
	CHRNB2	RECK	ARHGEF16	LTB4R
	EHMT1	RELA	GPR18	CCR10
	KMT2A	REN	CRTC3	FCN2
	SMARCE1	RETN	PHF19	NAGLU
	PFKFB3	RETNLB	MSI1	CYB5A
	PTPRN2	RGS2	NEK6	IRF4
	CCNB2	RGS4	POLD3	CRTC3
	APOA2	RHO	PPP2R3C	ADSL
	MPZ	RHOA	ZMYND8	CCND2
	LITAF	RIC1	ZFYVE26	USH1C
	LPP	RIPK2	TAX1BP1	C5AR1
	ID2	RNASE2	TDRKH	CCR1
	SF3B4	RNASE3	RNF144A	FGA
	PSMD4	RNASEH2C	SLC15A2	BCL2L11
	PTPRK	ROBO1	RASA2	HDAC2

ADAMTS4	ROM1	RBKS	TREM1
FOXC1	RORA	SLC25A36	MDC1
PRPF3	RORC	RALGAPA1	CHRM1
ZFP36L1	RPS27A	PPCDC	HMOX2
ARFGAP1	RPS28P1	SLC7A10	MICB
ATP6V0A1	RPTN	CGN	MMEL1
AFF4	RREB1	CDC23	ANKRD55
AHI1	RUNX1	BTN2A1	AKR1A1
EDEM1	RUNX3	B4GALT3	TNFSF4
ITPKA	RYR1	GNPDA1	MMP14
KPNA3	RYR2	GJA10	NMNAT1
CNTNAP1	S100A12	GPA33	EIF4A3
MYT1L	S100A7	NRBP1	DNMT3B
PA2G4	S100A8	NEGR1	TGFB3
LOXL4	S100A9	SCAMP2	CLN3
PRKRA	S100B	RGL2	DEFA5
TRAF1	S1PR1	THEM4	TNFRSF11B
TARS2	SCG3	SNX27	ERCC4
RNF5	SCGB1A1	ZNF217	GSN
RPL27	SCGB3A2	CREG1	CEBPB
PDCD6IP	SCN4A	BATF3	GALR1
RFX5	SCNN1A	DDX31	RARA
RAG2	SDCCAG8	COL15A1	FER
PSMD3	SDHB	DIDO1	S100A12
CHST8	SDHD	AKAP11	CYP27A1
ATP6V1D	SEC16B	BTN2A2	COL5A2
CYTH2	SEC24C	BNC2	HPX
CXCR5	SELE	CARD6	PHF6
ITGB8	SELL	ASCL2	IFT172
PEX13	SELP	GIMAP4	TMEM67
VTI1B	SELPLG	GIMAP5	TMEM216
VPS11	SEMA3A	EYA2	TIMP3
TREH	SEMA3E	IKZF4	PTPN11
CLASP2	SEMA4A	KLF12	FIP1L1
ATG4B	SEPT5-GP1BB	COG6	SLC19A3
AAGAB	SERPINA1	JAZF1	POU5F1
KDELR1	SERPINA3	GPN1	BDH2
PHF19	SERPINA6	HS3ST3B1	HNF1A-AS1
NUBP1	SERPINB1	PMEL	MUC1
TAX1BP1	SERPINB10	OTULIN	AK2
TDRKH	SERPINB2	NUB1	ETV6
SLC7A10	SERPINB3	OLIG3	RERE
CGN	SERPINB4	MAN2C1	CD300C
AOAH	SERPINB7	NDFIP1	PSMB8

B4GALT3	SERPINE1	LMAN2	CLEC4M
BTNL2	SERPINE2	PPT2	IGFBP4
FGF20	SETDB2	ZFP64	THBD
KLHL20	SFRP2	ZNF365	PTGER2
KANK2	SFRP5	TEF	TAS2R10
KCTD15	SFTA3	TSHZ2	PLA2G6
GPA33	SFTPA1	RPS25	PTCH1
RBM17	SFTPA2	SLC25A16	GJB2
ZNF217	SFTPB	NINL	ACKR2
VPS25	SFTPC	RAD51B	KLF5
AFF1	SFTPD	THEMIS	TM6SF2
CREG1	SH2B1	TM9SF2	ACP2
DBP	SH2B3	SPSB1	SMARCE1
JAZF1	SHH	SENP5	SMARCC2
PAPOLG	SIGIRR	TOB2	ADNP
NDFIP1	SIGLEC5	VAX2	MGRN1
PSMD5	SIGLEC8	ADO	IRAK3
UBE2Q1	SIK3	ARHGAP27	ADRA1B
TEF	SIM2	CDK2AP1	NLRC4
TPD52L2	SIRT1	ABO	LRP2
TRIB1	SIX3	BTG4	CIITA
TRIM39	SLC11A1	ATP10B	TIPARP
RNF111	SLC18A2	BUD13	KRT5
PGLYRP4	SLC22A2	BTN3A2	FECH
RAD51B	SLC22A3	CSDC2	ITGB1
SNRNP70	SLC22A4	FAM177A1	DPM1
TMOD4	SLC22A5	FNIP1	NPS
TOB2	SLC24A2	FKBPL	PSEN2
ARL8B	SLC24A3	DUSP12	SFPQ
CYTH4	SLC25A46	PLB1	TCAP
FNIP1	SLC26A4	NANP	AIF1
FBXW2	SLC26A9	MDN1	MIR1207
HORMAD1	SLC27A4	STX18	SEMA4D
KLHL8	SLC30A8	TCHH	EGR1
DUSP12	SLC44A1	TNFAIP8	RXRA
CNTRL	SLC5A12	SLC2A4RG	ENO1
FOXF2	SLC6A11	SLC25A33	
STMN3	SLC6A12	UCKL1	
RND2	SLC6A14	ZBTB38	
RASIP1	SLC6A4	ZNF687	
RTF1	SLC6A7	ZPR1	
NRBF2	SLC7A2	CASC3	
PIH1D1	SLC9A1	ATG9B	
SEMA6C	SLC9A3	GIMAP2	

ZGPAT	SLC9A3R1	GIMAP7
YTHDF1	SLPI	LAYN
VPS72	SMAD2	DPY19L3
CEP89	SMAD3	FOXR1
ALDH16A1	SMAD4	NIPSNAP3B
ABHD16A	SMAD6	MOB4
DPY19L3	SMAD7	MMEL1
H2AX	SMARCE1	PITPNM2
LY6G6C	SMOC2	KCNG1
MRPS21	SNW1	WDR46
MDM1	SNX9	WIPF2
UPK2	SOCS1	TRMT10C
RPRD2	SOCS2	UPK2
THEM5	SOCS3	THEM5
SPPL3	SOCS5	SNX24
RANBP6	SOD1	SPPL3
CELF3	SOD2	SCAMP5
COMMD9	SOD3	SDK1
BCL9L	SOX9	ZNF652
GAL3ST2	SP1	CCDC91
IZUMO1	SPATS2L	CELF3
DEDD	SPINK5	ABT1
GOLPH3L	SPINT2	BCL9L
MRPL41	SPP1	BTN3A3
RFTN2	SPRR2B	GIMAP6
SPEF2	SRC	GAL3ST2
CDC42SE1	SST	DEDD
BNIPL	ST2	SURF6
B3GALT1	STAT1	RPP25
ARRDC1	STAT2	VASH2
EBPL	STAT3	CLEC4F
GMEB2	STAT4	CACFD1
GID8	STAT5A	ANKRD46
NRROS	STAT5B	C15orf39
PRM2	STAT6	GIMAP1
PRRT1	STIN2-VNTR	EGFL8
ZNF507	STIP1	FBXO22
ZMYND19	STK33	LIME1
TCHHL1	STX1A	DCAF5
TPRG1	STXBP1	NGRN
SLC10A6	SUFU	NRROS
RCSD1	SULT1A1	MRPL9
PIGX	SULT1A3	MIER1
ZCCHC10	SUOX	PRM2

SLTM	SUZ12	NELFE
CNOT10	SVEP1	ZNF507
CEP19	SYK	WDR43
CAPSL	TAC1	TESPA1
GRWD1	TAC3	STARD6
IQCH	TAC4	TATDN3
MUCL1	TACR1	TCHHL1
P3H4	TACR2	TNP2
OAZ3	TAF7	TPRG1
LMBRD2	TAFA2	SLC9B1
ICE2	TAGLN	RCSD1
R3HCC1L	TALDO1	OGFOD2
RAPGEFL1	TAMM41	PIGX
TOMM40L	TAP1	THAP4
GHDC	TAP2	ZCCHC10
KRT222	TBCK	ZCCHC2
PLEKHH3	TBX1	ZPBP2
LYSMD1	TBX21	CEP19
KRTAP3-2	TBXA2R	C12orf43
MAMSTR	TBXT	CAPSL
LINGO4	TDGF1	DMXL1
PRR12	TEK	GPANK1
ZNF385C	TERT	FAM71A
ATP5MG	TF	FBXO45
C4orf36	TFAP2A	HMGN4
FAM71D	TFRC	MUCL1
DEXI	TG	PCDHB7
HSPB9	TGFA	OAZ3
NUP210L	TGFB1	LMBRD2
THAP12	TGFB2	TTC33
TDRD10	TGFB3	RAPGEFL1
CRCT1	TGFBR1	SETD4
IFTAP	TGFBR2	TOMM40L
CIART	TGFBR3	ZNF671
OTULINL	TGIF1	UBXN7
MINDY2	TGM1	GSAP
RPL41	TGM2	KRT222
TMEM235	TGM3	DCAF1
CFAP126	TGM5	GPATCH1
FDX2	TH2-LCR	LRRIQ3
LCE3E	THBD	PRR5L
HMSD	THPO	ZNF704
MUCL3	TIMD4	SUPT7L
CFAP141	TIMELESS	SMTNL2

CENATAC	TIMP1	SELENON
AHSA2P	TJP1	SLC45A1
LCE1E	TLE4	SPHKAP
TNXA	TLR1	ZNF512
LCE1F	TLR10	KRTAP3-2
SNHG32	TLR2	OBP2B
EGOT	TLR3	LINGO4
ADM5	TLR4	PXYLP1
IRF1-AS1	TLR5	ZDHHC12
MEIKIN	TLR6	ATP5MG
HNF1A-AS1	TLR7	H1-3
OIP5-AS1	TLR8	H1-6
MIR28	TLR9	ZNF322
HCG18	TMEM132D	TMPPE
LINC00298	TMEM18	H2BC5
JAZF1-AS1	TMEM79	DLEU1
SNORA31	TMPO	H4C3
LINC00861	TNC	LRRC43
MIR554	TNF	MTRFR
MAP3K14-AS1	TNFAIP1	ZNF774
PRKCQ-AS1	TNFAIP3	TEX33
SNORD16	TNFAIP8L2	PRM3
SNORD35A	TNFRSF10A	PHETA1
SNORD23	TNFRSF10B	CFAP126
SNORD35B	TNFRSF11B	FBH1
BHLHE40-AS1	TNFRSF13B	RELCH
C20orf181	TNFRSF18	MFSD13A
H2BC20P	TNFRSF1A	PRXL2B
LINC00624	TNFRSF1B	CENATAC
STEAP2-AS1	TNFRSF4	BTN2A3P
OR2W5P	TNFRSF6B	DELEC1
FOXCUT	TNFRSF8	STUM
ID2-AS1	TNFSF10	SPATA45
SEC1P	TNFSF12	MIR34B
XIRP2-AS1	TNFSF13B	FAM157A
CHROMR	TNFSF14	HSPE1-MOB4
C2CD4D-AS1	TNFSF4	MIR34C
MIR3936HG	TNS1	FAM182A
REL-DT	TNXB	HOATZ
ZBTB46-AS1	TOP2A	GABARAPL3
ENSG00000235978	TOR1B	IRF1-AS1
CUTALP	TP53	MEIKIN
AOC4P	TPMT	HNF1A-AS1

DCAF8-DT	TPO	OIP5-AS1
IL6R-AS1	TPSAB1	RTEL1-TNFRSF6B
H2BC19P	TPSB2	PCBP1-AS1
LINC01882	TPSD1	MIR4435-2HG
LINC02042	TPSG1	PPP1R2P1
UBE2Q1-AS1	TPT1	LINC00877
RPS3AP21	TRAIP	HOXA10-AS
SNORA58B	TREM1	LINC02912
CNOT10-AS1	TREX1	CROCCP2
AFF1-AS1	TRG	CROCCP3
LINC02648	TRIM26	CALML3-AS1
NOP53-AS1	TRIM32	DLG1-AS1
NBPF17P	TRPA1	JAZF1-AS1
MIR302F	TRPC1	GUSBP2
LINC01993	TRPC3	PITX1-AS1
LINC01143	TRPM8	ADAM1A
LINC01991	TRPV1	LINC00705
TDRKH-AS1	TRPV4	MIR554
USP34-DT	TSBP1	MIR647
NONOP2	TSLP	PRKCQ-AS1
RFX5-AS1	TTC8	STX18-AS1
PTPRK-AS1	TTLL1	SENCR
RPS10P6	TUSC3	ZNRD1ASP
RSL1D1-DT	TWIST1	FLVCR1-DT
ENSG00000167807	TXK	HCG25
CICP4	TXN	MIR3142HG
AKR1D1P1	UBE3C	SPRY4-AS1
LINC02863	UFD1	SDHAP2
NFILZ	UGT1A6	FAM172BP
SUGT1P2	UNC45A	LINC00393
RPS12P3	USB1	ESPNP
RNU6-850P	USP7	SDHAP1
RNU7-57P	UTS2	C2CD4D-AS1
RNU1-134P	VAV3	ETS1-AS1
RPS9P4	VCAM1	LINC01063
RAB5C-AS1	VCAN	DNM1P34
RN7SL688P	VDR	DNM1P35
RNU6-884P	VEGFA	CDRT8
ENSG00000232937	VIP	NCF4-AS1
ENSG00000234793	VPS51	LINC02240
ENSG000002025	VSIR	MIR3936HG

33		
ENSG00000224645	VTN	MIR623
ENSG00000226375	VWF	MELTF-AS1
ENSG00000233411	WAC	ZNF337-AS1
ENSG00000259623	WDPCP	WAKMAR2
ENSG00000267765	WDR19	LOC100506406
ENSG00000269621	WDR36	ATP6V1B1-AS1
ENSG00000252840	WDR46	AQP5-AS1
ENSG00000257870	WIF1	DCAF8-DT
ENSG00000259202	WNT5A	HLA-DQB3
ENSG00000238280	XDH	FOSL2-AS1
ENSG00000248969	XKR6	MUC20-OT1
ENSG00000250264	XPR1	MIR6731
BCLAF1P1	XRCC3	MIR4686
EEF1A1P27	ZAP70	LINC02098
LCEP4	ZBTB10	PITPNM2-AS1
PUDPP2	ZBTB38	PSMB8-AS1
SUMO2P10	ZDHHC24	SEMA6A-AS2
TECRP1	ZIC2	RPL21P124
RNU6-603P	ZMPSTE24	OR7E62P
RNU6-979P	ZNF331	LOC100130744
SEC13P1	ZNF365	LOC101929295
RNU7-97P	ZNF665	ITGB8-AS1
ENSG00000199332	ZNF699	LINC02648
ENSG00000224000	ZPBP2	LINC01991
ENSG00000267002		LINC02341
ENSG00000267520		LINC01149
ENSG000002848		TDGF1P2

29	
ENSG00000260773	TDRKH-AS1
ENSG00000272279	RPS17P2
ENSG00000284968	RERE-AS1
ENSG00000236427	RFX5-AS1
ENSG00000268093	PTPRK-AS1
ENSG00000229021	SDHAP4
DBF4P2	LOC100129603
RN7SL474P	LOC100996583
RNA5SP144	HLA-U
RN7SL352P	LINC02676
ENSG00000286587	LINC02773
lnc-KCNJ1-3	RPLP0P3
ENSG00000201680	RPS10P6
ENSG00000224431	SOCAR
lnc-FLI1-4	ENSG00000225643
lnc-SUMF1-4	ENSG00000249494
lnc-SUMF1-12	ENSG00000234084
ENSG00000255186	AKR1D1P1
ENSG00000273333	ALDH7A1P4
ENSG00000283782	ARF1P2
ENSG00000236864	CCR12P
ENSG00000240751	KRT8P46
ENSG00000273160	ETV7-AS1
lnc-CD247-1	LINC02863
lnc-PRKCQ-3	LINC02177
LARP7P4	MTCO3P1
GTF3AP1	LINC02571
ENSG000002869	LRRC37A15P

74	
lnc-IRF1-1	RPL31P12
ENSG00000287218	RN7SL688P
lnc-SUMF1-18	RPL32P23
lnc-SUMF1-19	RPS27P25
ENSG00000283411	RUNX3-AS1
lnc-BCL6-7	SNRPGP7
lnc-SMARCE1-2	YWHAQP6
lnc-SMARCE1-1	LOC101928272
lnc-SEMA6C-3	ZNF652-AS1
lnc-TRIB1-12	ENSG00000232937
lnc-ZMYND19-2	ENSG00000234793
piR-43107-023	ENSG00000227938
RF00017-5687	ENSG00000200753
SDR42E1P5	ENSG00000202533
RNU6-408P	ENSG00000224077
lnc-GPSM3-1	ENSG00000224374
lnc-HLA-DQA1-8	ENSG00000224645
lnc-IZUMO1-2	ENSG00000217455
lnc-LYSMD1-1	ENSG00000226375
lnc-ICE2-7	ENSG00000224269
lnc-IL1RL1-3	ENSG00000225744
lnc-HLA-DRB1-6	ENSG00000226645
lnc-IZUMO1-1	ENSG00000203392
lnc-KCTD15-5	ENSG00000221083
lnc-FOXC1-2	ENSG00000233411
lnc-STMN3-5	ENSG00000234389
lnc-NEMP1-2	LOC105369519
lnc-NOTCH4-1	LOC101927770
LOC105376934	LOC101927243
ENSG00000251411	ENSG00000236352
HSALNG0007192-001	ENSG00000238142
HSALNG0017424	ENSG00000251867
HSALNG0031446	ENSG00000255946
HSALNG0035673	ENSG00000260271

HSALNG0041154	ENSG00000262039
HSALNG0007401	ENSG00000244151
HSALNG0007916	ENSG00000250948
HSALNG0008492	ENSG00000255060
HSALNG0008504	ENSG00000269621
HSALNG0023888	ENSG00000270210
HSALNG0028061	ENSG00000272462
HSALNG0031449	ENSG00000250186
HSALNG0049384	ENSG00000252840
HSALNG0049385	ENSG00000254928
HSALNG0049386	ENSG00000254980
HSALNG0075913	ENSG00000256364
HSALNG0025043	ENSG00000256569
HSALNG0031953	ENSG00000257870
HSALNG0044846	ENSG00000259202
HSALNG0075447	ENSG00000248969
HSALNG0085865	ENSG00000248993
HSALNG0087584	ENSG00000250264
HSALNG0091393	ENSG00000254027
ENSG00000285458	ENSG00000270640
HSALNG0085864	ENSG00000228334
HSALNG0091590	ENSG00000229299

HSALNG0105356	ENSG00000229971
HSALNG0106356	ENSG00000230732
HSALNG0116381	ENSG00000232807
HSALNG0091400	AIMP1P2
HSALNG0123922	EEF1A1P27
HSALNG0126903	H2AC5P
HSALNG0131666	GAPDHP54
lnc-ANAPC1-3	PSMD10P3
lnc-ARFRP1-1	TRN-GTT4-1
lnc-ATP6V1G2-DDX39B-5	RLIMP1
AB372574	RN7SKP297
HE856132	RPL21P81
HSALNG0008232	RNU6-979P
HSALNG0012816	RNU7-130P
HSALNG0075965	RNU6-1228P
HSALNG0087578	RNA5SP443
HSALNG0087581	RNU1-150P
lnc-APOA2-2	SETP16
lnc-ATP6V0A1-5	RANP3
lnc-CAVIN1-1	ENSG00000286863
lnc-SLTM-2	ENSG00000235862
lnc-SMARCE1-4	ENSG00000287937
lnc-TMEM235-1	ENSG00000201451
lnc-TMPPE-1-001	ENSG00000206649
lnc-TMPPE-1-002	ENSG00000199867
lnc-ZGLP1-1	ENSG00000223643
lnc-TMPPE-3	ENSG00000223808
lnc-TNFSF18-4	ENSG00000223837

MN298214	ENSG00000222529
NONHSAG043568.2	LOC101928279
OA985677	ENSG00000253238
piR-31470-402	ENSG00000255605
piR-31937-039	ENSG00000262020
piR-38051-185	ENSG00000285016
piR-39701-037	ENSG00000245869
MK280221-009	ENSG00000259177
NONHSAG045731.2	ENSG00000231324
piR-38351-029	RN7SL474P
piR-51710	ENSG00000286518
MK280269-004	ENSG00000287023
NONHSAG034319.2	ENSG00000288542
piR-33614-243	lnc-MAP3K7-3
piR-38959-011	lnc-MEGF6-3
piR-49732-033	lnc-IL1A-3
MN308805	lnc-MPHOSPH9-2
piR-33458	TREHP1
piR-36756-025	ENSG00000197670
piR-61289-077	ENSG00000224431
RF00017-306	lnc-FOSL2-2
RF00026-971	ENSG00000235241
RF00017-2701	ENSG00000272162
RF00017-310	ENSG00000274092
RF00017-3884	ENSG00000280878
RF00017-960	ENSG00000237371
piR-59241	ENSG00000283782
RF00017-2197	ENSG00000260274
RF00017-4442	lnc-CDC42BPA-5
lnc-KCTD15-4	lnc-FAM76B-1
lnc-MERTK-5	ENSG00000236864
HSALNG0083655	ENSG00000273160
HSALNG0083656	lnc-CD247-1
LOC112267902	lnc-CEP19-1
LOC112268240	ENSG00000228037
ENSG00000218027	lnc-PRKCQ-3
LOC102723944	lnc-TNFSF18-1
LOC105371434	lnc-TNFAIP3-1

LOC105372675	lnc-VPS37C-3
LOC105370790	RF00017-7460
LOC101928512	IFITM3P5
HSALNG0079998	THAP12P4
HSALNG0007917	ENSG00000285842
HSALNG0023718	lnc-MMEL1-1
HSALNG0044120	lnc-HLA-DMA-2
HSALNG0044122	lnc-HLA-DQB1-2
HSALNG0047398	lnc-IRF1-1
HSALNG0047400	LOC107986649
HSALNG0056490	lnc-FLI1-3
HSALNG0015261	ENSG00000287218
HSALNG0015265	ENSG00000254851
HSALNG0023719	ENSG00000255093
HSALNG0049257	ENSG00000277938
HSALNG0049431	ENSG00000285551
HSALNG0097072	HSALNG0000677
HSALNG0106269	lnc-CARD11-5
HSALNG0116307	lnc-CREB5-4
HSALNG0116509	lnc-ETS1-8
HSALNG0123786	lnc-FAM109A-5
HSALNG0130979-002	ENSG00000239783
HSALNG0130979-003	L13304-010
HSALNG013166	lnc-ABO-33

9	
HSALNG0007020-001	lnc-AKR1C2-3-002
HSALNG0015266	lnc-BCL6-7
HSALNG0017416	HSALNG0135257
HSALNG0017893	lnc-APOA1-4
HSALNG0025039	lnc-DTWD2-15
HSALNG0068533	lnc-SMARCE1-2
HSALNG0078332	lnc-OR13C9-2
HSALNG0106270	lnc-SMARCE1-1
HSALNG0116510	lnc-POLD3-7
HSALNG0123985	lnc-PXYLP1-3
lnc-PGLYRP4-1	lnc-RPP25-3
lnc-PRR5L-1	lnc-TEF-1
MK280073-186	lnc-TFDP2-12
MN309005	lnc-UBAC2-4
piR-32810-041	lnc-VWA8-7
piR-45578	NONHSAG043563.2
piR-48918	NONHSAG018964.2
piR-48325-029	piR-43107-023
piR-48772-016	RF00017-1167
MN298065	LOC100128721
piR-41525-585	ENSG00000285837
piR-53338-015	lnc-GJA10-23
RF00017-2367	lnc-ITGB8-5
RF00017-3643	lnc-LPP-8
piR-57394-217	lnc-GATA3-19-001
RF00017-287	lnc-GATA3-19-002
piR-56497-066	lnc-GATA3-20
piR-59907-003	lnc-KLF5-11
RF00017-2185	lnc-KIN-10
RF00017-2956	LOC107986589
piR-57197-002	LOC105376805

piR-58029-003	lnc-NDFIP1-1
RF00917	LOC105377891
HSALNG0123784	LOC105369325
piR-32023-024	lnc-SUFU-1
piR-42613-003	LOC105373023
piR-31162-048	LOC105372579
piR-55654-049	LOC100216346
piR-56451-016	LOC105369308
piR-59316-009	LOC105374724
IL2RG	ENSG00000254478
ACTRT1	ENSG00000249626
ACVRL1	ENSG00000285552
SH2B3	HSALNG0007192-001
BCL3	HSALNG0013864
RASA2	HSALNG0031444
SDC4	HSALNG0031446
ORM1	HSALNG0033906
ATOD4	HSALNG0036089
ATOD2	HSALNG0036090
CARMA1	HSALNG0036091
BIMP3	HSALNG0004423
PPBL	HSALNG0007916
BENTA	HSALNG0008504
IMD11A	HSALNG0022773
	HSALNG0031449
	HSALNG0041149
	HSALNG0073935
	HSALNG0075913
	FJ601684-088
	HSALNG0031953
	HSALNG0032010-001
	HSALNG0044846
	HSALNG0049430
	HSALNG0062314
	LOC105378797
	HSALNG0087584
	HSALNG0091393
	HSALNG0109576
	lnc-ATP10B-2
	lnc-C15orf39-2
	lnc-CDK2AP1-1

lnc-DNAJB7-2
lnc-ERRFI1-4
lnc-FAM109A-1
ENSG00000279322
ENSG00000285616
HSALNG0085720
HSALNG0091590
HSALNG0105356
HSALNG0116381
HSALNG0130934
L13304-032
L13715-023
lnc-ANGPTL1-5
lnc-BATF3-4
HSALNG0088091-001
HSALNG0088091-002
HSALNG0088092
HSALNG0088094
HSALNG0091400
HSALNG0130835
HSALNG0130838
HSALNG0130833-002
L13713-173
lnc-ADGRD2-2
lnc-AKR1C2-3-001
lnc-BRAP-1
AB372574
HE855948
HE856132
HSALNG0008232
HSALNG0017888
HSALNG0026811
HSALNG0026812
HSALNG0049436
HSALNG0059112
HSALNG0066495
HSALNG0073211
HSALNG0075965
HSALNG0078328
HSALNG0087578
HSALNG0087581

L13304-025
L13715-019
lnc-APOA2-2
lnc-PADI1-5
lnc-RAB5B-2
lnc-SLC15A3-2
lnc-POP5-3
lnc-PPCDC-1
lnc-SMARCE1-4
lnc-TNFRSF14-3
lnc-ZBTB10-4
lnc-TTC33-6
lnc-TLR1-1
lnc-TFDP2-13
lnc-TNFSF18-4
lnc-TOR3A-1
MN296792
MN298214
piR-35516
piR-51137-089
piR-38351-029
piR-42324
piR-46002-146
piR-50443-559
piR-32214-183
piR-33614-024
piR-49732-033
MN296981
MN297833
NONHSAG009596.
2
NONHSAG024039.
2
NONHSAG031908.
2
NONHSAG041785.
2
NONHSAG045982.
2
piR-33458
piR-39701-054
piR-45012-054
piR-48553-160
piR-50437-440

piR-59591
piR-61289-077
RF00017-1218
RF00017-306
RF00017-4535
RF00017-6730
RF00026-971
piR-59218
RF00017-1685
RF00017-2701
RF00017-4513
RF00017-949
piR-55194-195
piR-57133-490
piR-59907-030
piR-60146-129
RF00017-029
RF00017-3884
RF00017-511
RF00017-5575
RF00017-7611
RF00017-847
RF00017-960
piR-59241
LOC102723444
piR-53096
LOC105376374
LOC105378327
LOC105371081
LOC105370790
LOC105371498
LOC105373117
LOC101928512
LOC105375130
LOC107984408
LOC107985101
LOC105376375
LOC105377347
ENSG00000260152
ENSG00000253237
HSALNG0015837
HSALNG0045165
HSALNG0053673
HSALNG0007917

HSALNG0023718
HSALNG0032006
HSALNG0040324
HSALNG0056490
HSALNG0010381
HSALNG0010382
HSALNG0010383
HSALNG0023719
HSALNG0032557
LOC105379185
LOC107986073
HSALNG0087119
HSALNG0087120
HSALNG0088729
HSALNG0114758
HSALNG0121975
HSALNG0125418
HSALNG0129374
KR153194-030
HSALNG0085719
HSALNG0085721
HSALNG0107248
HSALNG0114031
HSALNG0091478
HSALNG0107249
HSALNG0129375
HSALNG0131670
HSALNG0131671
HSALNG0025039
HSALNG0049449
HSALNG0066497
HSALNG0066501
HSALNG0073210
HSALNG0109575
HSALNG0109581
lnc-ZNF286A-13
lnc-ZPBP2-3
MN309005
piR-38580-086
piR-38716-085
piR-40352
piR-43106-052
piR-32382
piR-36588-004

piR-38872-001
piR-42491-196
MN309174-433
piR-36362-005
piR-37170-024
piR-37560
piR-38216
piR-44855
piR-48799-021
piR-50437-154
NONHSAG031521.
2
piR-36365-003
piR-36365-004
piR-39488-281
piR-50136
piR-52473
piR-54025
RF00017-1201
RF00017-3552
RF00017-4542
RF00017-4592
RF00017-7459
RF00017-7356
piR-56905
piR-57460-460
piR-58297-114
RF00017-6733
RF00017-6735
RF00994-270
piR-53412
piR-58557-051
piR-59258
RF00026-1060
RF00994-258
LOC107984976
HSALNG0114759
HSALNG0049448
piR-32023-024
piR-42613-003
ESR2
STK11
C4B
MKI67

MIR1-1
PLAUR
SERPINB2
CTNNB1
XCR1
ADRA1D
BHR1
