# Supplementary material for: Network Pharmacology and Molecular Docking Study of Yupingfeng Powder in the Treatment of Allergic Diseases
Source: Evid Based Complement Alternat Med. 2022 Jul 9;2022:1323744. doi: 10.1155/2022/1323744 (PMC9288288; doi:10.1155/2022/1323744)
Supplement: Supplementary Materials — Supplementary Table S1: YPFP-related target genes obtained by TCMSP target gene prediction and UniProt gene name transformation. Supplementary Table S2: target genes corresponding to 5 keywords of “atopic dermatitis,” “atopic eczema,” “asthma,” “allergic rhinitis” and “food allergy.” Supplementary Table S3: node degree of each protein in PPI network. Supplementary Table S4: top 10 in the PPI network ranked by the MCC method. Supplementary Table S5: PDB IDs and references of key proteins. [file 1323744.f1.zip › Supplementary Table S3.pdf]

Supplementary Table S3

Node degree of each protein in PPI network.

node	identifier	node_degree
IL6	9606.ENSP00000385675	45
TNF	9606.ENSP00000398698	44
IL1B	9606.ENSP00000263341	43
TP53	9606.ENSP00000269305	43
PTGS2	9606.ENSP00000356438	42
CXCL8	9606.ENSP00000306512	41
VEGFA	9606.ENSP00000478570	41
JUN	9606.ENSP00000360266	39
PPARG	9606.ENSP00000287820	39
CCL2	9606.ENSP00000225831	38
IL10	9606.ENSP00000412237	38
MMP9	9606.ENSP00000361405	38
IFNG	9606.ENSP00000229135	37
IL4	9606.ENSP00000231449	37
NFKBIA	9606.ENSP00000216797	36
EGFR	9606.ENSP00000275493	35
ICAM1	9606.ENSP00000264832	35
VCAM1	9606.ENSP00000294728	35
HMOX1	9606.ENSP00000216117	34
IL2	9606.ENSP00000226730	34
MMP2	9606.ENSP00000219070	34
CRP	9606.ENSP00000255030	32
MPO	9606.ENSP00000225275	32
TGFB1	9606.ENSP00000221930	32
ESR1	9606.ENSP00000405330	31
IL1A	9606.ENSP00000263339	31
CXCL10	9606.ENSP00000305651	30
NOS3	9606.ENSP00000297494	30
MMP1	9606.ENSP00000322788	28
NOS2	9606.ENSP00000327251	28
SELE	9606.ENSP00000331736	28
ERBB2	9606.ENSP00000269571	27
MAPK14	9606.ENSP00000229795	26
CD40LG	9606.ENSP00000359663	23
AHR	9606.ENSP00000242057	21
IRF1	9606.ENSP00000245414	19
ALOX5	9606.ENSP00000363512	18
AR	9606.ENSP00000363822	18
DPP4	9606.ENSP00000353731	18
MAPK1	9606.ENSP00000215832	18
PLAU	9606.ENSP00000361850	18

CYP1A1	9606.ENSP00000369050	15
GSTP1	9606.ENSP00000381607	14
CYP3A4	9606.ENSP00000337915	13
PTGS1	9606.ENSP00000354612	13
ADRB2	9606.ENSP00000305372	10
GSTM1	9606.ENSP00000311469	9
SLC6A4	9606.ENSP00000261707	4
