# Supplementary material for: Network Pharmacology and Molecular Docking Study of Yupingfeng Powder in the Treatment of Allergic Diseases
Source: Evid Based Complement Alternat Med. 2022 Jul 9;2022:1323744. doi: 10.1155/2022/1323744 (PMC9288288; doi:10.1155/2022/1323744)
Supplement: Supplementary Materials — Supplementary Table S1: YPFP-related target genes obtained by TCMSP target gene prediction and UniProt gene name transformation. Supplementary Table S2: target genes corresponding to 5 keywords of “atopic dermatitis,” “atopic eczema,” “asthma,” “allergic rhinitis” and “food allergy.” Supplementary Table S3: node degree of each protein in PPI network. Supplementary Table S4: top 10 in the PPI network ranked by the MCC method. Supplementary Table S5: PDB IDs and references of key proteins. [file 1323744.f1.zip › Supplementary Table S4.pdf]

Supplementary Table S4

Top 10 in PPI network ranked by MCC method.

**Rank**	**Name**	**Score**
1	IL6	8.59E+22
1	TNF	8.59E+22
3	IL1B	8.59E+22
4	CCL2	8.59E+22
5	PTGS2	8.59E+22
6	CXCL8	8.59E+22
7	IL10	8.59E+22
8	IL4	8.59E+22
9	JUN	8.59E+22
10	IFNG	8.59E+22
