# Supplementary material for: Network Pharmacology and Molecular Docking Study of Yupingfeng Powder in the Treatment of Allergic Diseases
Source: Evid Based Complement Alternat Med. 2022 Jul 9;2022:1323744. doi: 10.1155/2022/1323744 (PMC9288288; doi:10.1155/2022/1323744)
Supplement: Supplementary Materials — Supplementary Table S1: YPFP-related target genes obtained by TCMSP target gene prediction and UniProt gene name transformation. Supplementary Table S2: target genes corresponding to 5 keywords of “atopic dermatitis,” “atopic eczema,” “asthma,” “allergic rhinitis” and “food allergy.” Supplementary Table S3: node degree of each protein in PPI network. Supplementary Table S4: top 10 in the PPI network ranked by the MCC method. Supplementary Table S5: PDB IDs and references of key proteins. [file 1323744.f1.zip › Supplementary Table S5.pdf]

Supplementary Table S5

PDB IDs and references of key proteins.

Protein	PDB ID	References
IL6	5FUC	https://www.rcsb.org/structure/5FUC
TNF	6CGI	https://www.rcsb.org/structure/6CGI
IL1B	1TWN	https://www.rcsb.org/structure/1TWN
CCL2	4ZK9	https://www.rcsb.org/structure/4ZK9
PTGS2	5F19	https://www.rcsb.org/structure/5F19
CXCL8	4XDX	https://www.rcsb.org/structure/4XDX
IL10	1ILK	https://www.rcsb.org/structure/1ILK
IL4	1HZI	https://www.rcsb.org/structure/1HZI
JUN	3PTG	https://www.rcsb.org/structure/3PTG
IFNG	1EKU	https://www.rcsb.org/structure/1EKU
